# Novel Disease Susceptibility Factors for Fungal Necrotrophic Pathogens in Arabidopsis

**DOI:** 10.1371/journal.ppat.1004800

**Published:** 2015-04-01

**Authors:** Albor Dobón, Juan Vicente Canet, Javier García-Andrade, Carlos Angulo, Lutz Neumetzler, Staffan Persson, Pablo Vera

**Affiliations:** 1 Instituto de Biología Molecular y Celular de Plantas, Universidad Politécnica de Valencia-C.S.I.C, Valencia, Spain; 2 Max Planck Institute of Molecular Plant Physiology, Golm/Potsdam, Germany; 3 ARC Centre of Excellence in Plant Cell Walls, School of Botany, University of Melbourne, Victoria, Australia; University of California, Davis Genome Center, UNITED STATES

## Abstract

Host cells use an intricate signaling system to respond to invasions by pathogenic microorganisms. Although several signaling components of disease resistance against necrotrophic fungal pathogens have been identified, our understanding for how molecular components and host processes contribute to plant disease susceptibility is rather sparse. Here, we identified four transcription factors (TFs) from Arabidopsis that limit pathogen spread. Arabidopsis mutants defective in any of these TFs displayed increased disease susceptibility to *Botrytis cinerea* and *Plectosphaerella cucumerina*, and a general activation of non-immune host processes that contribute to plant disease susceptibility. Transcriptome analyses revealed that the mutants share a common transcriptional signature of 77 up-regulated genes. We characterized several of the up-regulated genes that encode peptides with a secretion signal, which we named PROVIR (for provirulence) factors. Forward and reverse genetic analyses revealed that many of the PROVIRs are important for disease susceptibility of the host to fungal necrotrophs. The TFs and PROVIRs identified in our work thus represent novel genetic determinants for plant disease susceptibility to necrotrophic fungal pathogens.

## Author Summary

Diseases caused by pathogens affect most plants in their natural environment. Plants combat the majority of these intruders by activating elaborate immune responses, which typically result in a disease resistance response. Nevertheless, pathogens have typically evolved ways to bypass plant defenses, and susceptibility to pathogens re-appears. In addition to this occasional immune failure of the host, other immune-response independent processes allow further ingress of the invading pathogen, and contribute to plant pathogen susceptibility. We identified four transcription factors (TFs) that are required for effective disease resistance to fungal necrotrophs. These TFs regulate critical aspects of disease resistance/susceptibility to necrotrophs without interfering with immune signaling. Mutant plants defective in any of these four TFs commonly induce about 80 genes, with a substantial proportion encoding peptides with secretion signals that are described to act as local signals (peptide hormones) during cell-to-cell communication, and that function in the plant-specific paracrine/autocrine system. We further show that many of these peptide hormones affect disease susceptibility of the host to necrotrophs. Our findings have thus uncovered many new factors that underpin immune-response independent processes of plant disease susceptibility.

## Introduction

Plants inhabit environments rich in microbial pathogens, which pose continuing threats to plant survival. However, very few pathogens are capable of successfully colonizing a specific host, suggesting the existence of efficient recognition mechanisms to activate plant defenses. In general, two types of microbial pathogens, which differ grossly in lifestyles, may be distinguished [1]. Necrotrophic pathogens use destructive and virulent strategies that promote cell death to acquire nutrients for their growth and reproduction [2,3]. This lifestyle contrasts with that of biotrophic pathogens, which require living plant cells for growth and reproduction. Both types of pathogens elicit distinct host immune responses.

Irrespective of pathogen type, the host perceives pathogens via plant pattern recognition receptors (PRRs) as pathogen-associated molecular patterns (PAMPs), and damage-associated molecular patterns (DAMPs) [4]. PAMPs are molecular tags essential for microbe viability, which are conserved between diverse genera, and are an efficient form of pathogen monitoring in plants. DAMPs are plant-generated signals in response to pathogen damage. PAMP recognition by PRRs triggers basal defense responses, known as pattern-triggered immunity (PTI). PTI provides protection against non-host pathogens, and limits disease caused by virulent pathogens [5]. The timing and efficiency in activating various basal defense mechanisms is thought to underlie differences in host susceptibility to necrotrophic pathogens.

Our understanding of PAMP-triggered necrotrophic immunity is largely derived from *Botrytis cinerea*; one of the most important fungal plant pathogens [2,3]. This species exhibits a broad geographic range and has the capacity to cause severe damage on plants, resulting in large economic losses in agriculture [6]. The fungal cell wall component chitin, and its constituent oligosaccharides, are fungal PAMPs, which activate numerous defense responses. Polygalacturonase (PG) is another fungal component essential for virulence. This protein is detected, independently of its enzymatic activity, by the plant as a PAMP and activates host defense responses [7]. Moreover, PGs can act on the host cell wall to degrade pectin, which is the primary carbon source for the pathogen, to produce oligogalacturonides (OGs). OGs of a certain length (10 to 15 degrees of polymerization) are, in turn, enriched by the activation of a plant encoded PG-inhibiting protein. These OGs may function as DAMPs that activate plant immunity [8]. A plant cell wall-associated kinase (WAK1) functions as a receptor for the immunoactive OGs [9]. In addition, intracellular mitogen-activated protein (MAP) kinase activity [10], and the CERK1 interacting receptor-like kinase (BIK1), appear essential to drive chitin induced PTI [11–13].

Resistance and susceptibility to necrotrophs is a genetically complex process and comprise the coordinated action of a wide range of hormones, including ethylene (ET), jasmonic acid (JA), salicylic acid (SA), and abcisic acid (ABA) [14–17]. In addition, secondary metabolites, including the phytoalexin camalexin, and other metabolites derived from tryptophan metabolism (e.g., indolglucosinolates), contribute to necrotroph resistance [18–20]. Resistance to necrotrophs is also determined by plant cell wall composition; mutants with defects in primary (e.g., *cev1* mutant) and secondary (e.g., *irx* mutants) wall cellulose synthase (CESA) expression or function showed increased resistance to necrotrophic fungi [21–24].

Coordinated and timely regulation of genes in immune response pathways is central to effective plant defense [25]. Different transcription factors (TFs) that affect immune responses towards necrotrophic fungal pathogens have been described: WRKY33 controls camalexin biosynthesis and is a major immune response regulator against necrotrophic fungi [26,27]; the MYB-related BOS1 restricts necrosis [28]; ASYMMETRIC LEAVES (AS1) regulates JA-related genes, which affects resistance against *B*. *cinerea* [29]; MYB51 is associated with the activation of indole glucosinolate biosynthetic genes [30]; the basic helix-loop-helix leucine zipper TF MYC2 regulates necrotroph resistance by modulating JA responses via an intermediate TF tier [31], a process antagonized by the ERF-type TF ERF1; the homeodomain OCP3, initially identified as a plant immunity repressor to necrotrophs [32], was recently shown to function as a regulator of an editing control mechanism of plastidial mRNAs [33]; and MYB46, which suppresses *B*. *cinerea* resistance by the regulation of genes encoding cell wall proteins and enzymes [23]. Interestingly, the enhanced disease resistance to *B*. *cinerea* in the *myb46* mutant was associated with an early down-regulation of *CESA* genes following fungal infection [34]. In summary, these results reinforce the hypothesis that a cell wall integrity surveillance system evolved to sense the presence of a pathogen, and to transduce signals into a rapid transcriptional reprogramming of the affected cell. This transcriptional reprogramming might serve to promote fungal growth or, alternatively, activate plant immunity, to facilitate susceptibility or resistance, respectively. The balance between these two responses might determine the degree of disease.

Although several molecular components that affect resistance or susceptibility against fungal necrotrophs have been characterized, identification of plant disease susceptibility factors that aid pathogens in gaining access to the host plant cell, or that facilitate fungal growth, remains limited. Here, we identify and characterize four TFs that are important for disease resistance towards two fungal necrotrophs, i.e. *Botrytis cinerea* and *Plectosphaerella cucumerina*. We subsequently identified a set of 77 genes that were commonly up-regulated in the four TF mutants. Many of these genes encode peptides that contain secretion signals that we tentatively have named PROVIR factors. We show that many of the PROVIR factors also function in disease susceptibility to fungal necrotrophic pathogens. Finally, *in vivo* localization studies revealed common pericellular localization of the PROVIR factors.

## Results

### Identification of transcription factorss co-regulated with *MYB46* and *CESA* genes

We sought to identify TFs that might mediate disease resistance and/or susceptibility towards fungal necrotrophs in Arabidopsis, in a manner similar to that described for MYB46. MYB46 was initially identified as a TF required for secondary cell wall formation in the vasculature of Arabidopsis [35]. Recently, Ramírez et al. [23] determined that MYB46 could function as a disease susceptibility modulator by controlling cell wall remodeling [23, 34]. In fact, *CESA* gene expression was transiently down-regulated following fungal inoculation, and this induced repression was more prominent in a *myb46* mutant [34]. These results therefore suggest that *MYB46* and *CESA* genes are interrelated in fungal disease resistance. In addition, *MYB46* and secondary wall *CESA* genes are transcriptionally coordinated, and in fact, transcriptionally coordinated genes, or co-expressed genes, tend to be functionally related [36]. We used the AraGenNet platform (http://aranet.mpimp-golm.mpg.de/) [37] to identify vicinity networks, i.e. co-expression neighborhoods, of genes co-expressed with *MYB46* (S1 Fig). Interestingly, this network vicinity also included genes encoding AtCESA7, AtESA8, and AtCESA4, further supporting a functional relationship of these proteins with MYB46.

We subsequently hypothesized that genes co-expressed with *MYB46* and *CESA4/7/8*, or genes in neighboring network vicinities, i.e. genes that are co-expressed with *MYB46* and the *CESA*s, might be of relevance to disease resistance and/or susceptibility to fungal necrotrophs. Based on functional annotation using MapMan ontology terms (http://aranet.mpimp-golm.mpg.de/; http://mapman.mpimp-golm.mpg.de/general/ora/ora.shtml), we identified genes encoding TFs and ordered T-DNA insertion lines for the TFs for which lines were available. A total of 18 genes encoding TFs of different types were finally selected for further study (see Table 1).

**Table 1:** List of genes encoding the selected TFs.

Gene locus	Protein name	T-DNA line	Description
At1g66810		SAIL_159_C03	Encodes a DNA binding protein, Zinc finger C-x8-C-x5-C-x3-H type family protein
At4g29080	PAP2/IAA27	SALK_070738.23.50	Aux/IAA-type transcription factor
At5g65320	BHLH99	SAIL_668_G04	(basic helix-loop-helix (bHLH) DNA-binding superf
At5g57520	ZFP2	SALK_007343.51.60	At5g57520 (Encodes a zinc finger protein containing only a single zinc finger.
At3g61910	NST2	SALK_142837.40.30	(NAC transcription factor regulating secondary wall thickening in anther walls
At5g25830	GATA12	SALK_112752.51.40	Encodes a member of the GATA factor family of zinc finger tran
At3g49930		SALK_093290.16.90	C2H2 and C2HC zinc fingers superfamily
At1g62360	STM	SAIL_402_D12	Class I knotted-like homeodomain protein that is required for shoot apical meristem
At4g01680	MYB55	SALK_087889.49.00	MYB-type transcription factor
At1g17950	MYB52	SALK_145442.22.25	MYB-type transcription factor
At2g38090		SALK_015863.41.65	homeodomain-like superfamily protein)
At5g16600	MYB43	SALK_085312.40.35	MYB-type transcription factor
At5g62380	VND6	SAIL_813_C12	Encodes a NAC-domain transcription factor involved in xylem formation
At1g12260	VND4	SALK_058195.50.00	Encodes a NAC-domain transcription factor. Expressed in vascular tissues
At2g44745	WRKY12	SALK_127750.42.20	WRKY-type transcription factor
At4g39410	WRKY13	SALK_032911.55.50	WRKY-type transcription factor
At1g74660		SAIL_789_A11	Encodes MINI ZINC FINGER 1 (MIF1) which has a zinc finger domain but lacks other protein motifs normally present in transcription factors
At5g18090		SALK_098295.53.00	AP2/B3-like transcriptional factor family

The genes in Table 1 were selected on the basis of co-expression in gene vicinity networks for *MYB46* and *CESA7*, *CESA8* and *CESA4*. Locus, common name, T-DNA insertion line used for functional studies, and description of structural domains or type of DNA binding domain found in each TF are indicated. T-DNA insertion mutants in the selected group of genes were further characterized in their response towards *B*. *cinerea* and *P*. *cucumerina*.

### Mutations in several of the selected transcription factors led to altered disease resistance against the necrotrophic fungal pathogens *P*. *cucumerina* and *B*. *cinerea*

**Fig 1:**
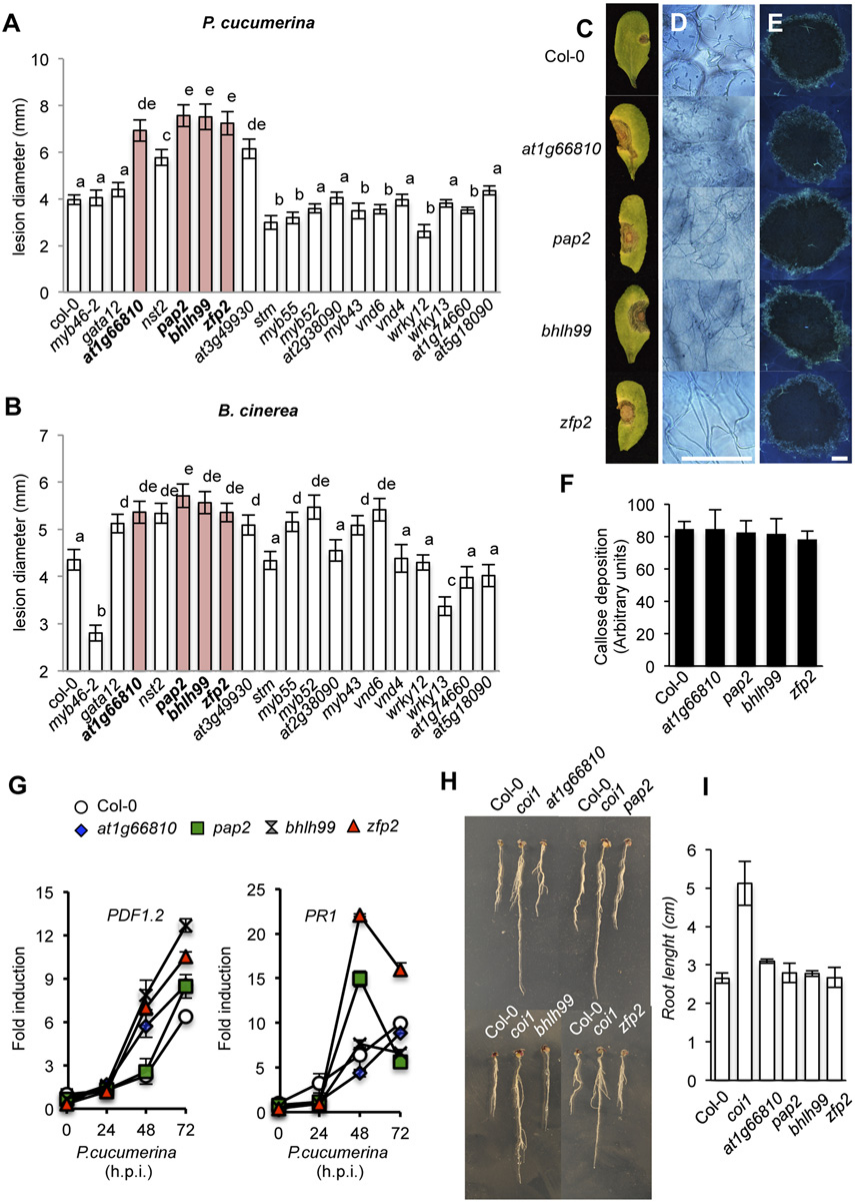
Characterization of disease resistance response of 18 mutants defective in the different transcription factors (TFs) identified as co-regulated with *MYB46* and *CESA4*, *CESA7* and *CESA8*. (**A-B**) Resistance response to *P*. *cucumerina* (**A**) and *B*. *cinerea* (**B**) in Col-0 and TF mutant plants evaluated 10 d post inoculation by determining average lesion diameter on four leaves per plant and from 15 plants per genotype. Data points represent average lesion size ± SE of measurements. An ANOVA was conducted to assess significant differences in disease symptoms, with a priori *P* < 0.05 level of significance; the letters above the bars indicate different homogeneous groups with statistically significant differences. (**C**) Representative leaves from Col-0, *zfp2*, *bhlh99*, *pap2*, and *at1g66810* plants at 10 d post inoculation with a 6-μL droplet of spores (2 x 10^4^ conidia mL^-1^; as in **A**) of *P*. *cucumerina*. (**D**) Trypan blue staining, at 72 h postinoculation (h.p.i) with *P*. *cucumerina*, shows increased proliferation and growth of fungal hyphae in the four mutants when compared to Col-0. (**E**) Aniline blue staining and epifluorescence microscopy was applied to visualize callose accumulation. Micrographs showing callose deposition following *P*. *cucumerina* infection in Col-0 and the four mutant plants at 48 h.p.i. Scale bars represent 500 μm. (**F**) Pathogen-induced callose deposition was calculated as arbitrary units by quantifying the number of yellows pixels per million on digital micrographs of infected leaves at 48 hpi. Bars represent mean ± SD, n = 15 independent replicates. (**G**) *PDF1*.*2* and *PR-1* expression in Col-0 and in the disease susceptible mutants *zfp2*, *bhlh99*, *pap2*, and *at1g66810* in early *P*. *cucumerina* infection stages. Relative expression was assayed over a 72-h time course by quantitative RT-PCR on total RNA from leaves following inoculation with a drop of spore suspension of *P*. *cucumerina*. Data represent means ± SD (n = 3 biological replicates). Expression was normalized to the constitutive *ACT2* gene. (**H-I**) *zfp2*, *bhlh99*, *pap2*, and *at1g66810* mutants, wild-type (Col-0) and *coi1* sensitivity to JA. Seedlings were grown for 7 days on agar plates supplemented with 50 μM JA. Root length reduction, diagnostic of sensitivity to JA, revealed no differences between the TF mutants and Col-0. The *coi1-40* mutant was insensitive to the hormone.

We next addressed whether or not the selected T-DNA mutants corresponding to the 18 TFs identified above were altered in their disease resistance to necrotrophs. We tested this by inoculating homozygous mutant plants with *B*. *cinerea* or *P*. *cucumerina*, and scored infected plants for disease symptoms by assessing the necrotic lesions in inoculated leaves (Fig 1). Wild-type (Col-0) and *myb46* mutant plants were used as negative and positive controls, respectively. As expected, Col-0 plants were susceptible to both pathogens, while *myb46* mutant plants showed increased resistance to *B*. *cinerea* (Fig 1A and 1B). In contrast, *at1g66810*, *nst2*, *pap2*, *bhlh99*, *zfp2*, and *at3g49930* insertion mutant plants exhibited enhanced disease susceptibility to both pathogens, and *gata12*, *myb55*, *myb52*, *myb43*, and *vnd6* insertion mutant plants showed enhanced susceptibility to *B*. *cinerea*, but not to *P*. *cucumerina* (Fig 1A and 1B).

### *zfp2*, *bhlh99*, *pap2* and *at1g66810* mutants are not defective in immune signaling

Since the *at1g66810*, *pap2*, *bhlh99* and *zfp2* insertion mutants exhibited strong susceptibility phenotypes to both *B*. *cinerea* and *P*. *cucumerina* they were selected for further characterization. At1g66810 belongs to a zinc finger C-x8-C-x5-C-x3-H type family of DNA binding proteins of unknown function and shows homology to CDM1 (CALLOSE DEFECTIVE MICROSPORE1), which is important for callose metabolism during microsporogenesis [38]. PAP2 is an Aux/IAA-type (IAA27) transcriptional repressor, and its homolog in *Medicago truncatula* is involved in arbuscular mycorrhizal symbiosis [39]. bHLH99 belongs to a basic helix-loop-helix DNA-binding superfamily of unknown function. ZFP2 is a zinc finger protein of unknown function, but phenotype data from over-expression studies suggest that ZFP2 participates in processes that directly or indirectly influence organ shedding [40]. The mutants affecting these four genes demonstrated characteristic necrotic extension upon inoculation with *P*. *cucumerina* (Fig 1C), followed by widespread proliferation of fungal mycelia, as revealed by trypan blue staining (Fig 1D). Callose deposition at the inner surface of epidermal cell walls is one of the earliest plant responses to fungal attack. To assess callose deposition in the inoculated leaves of the four mutants and Col-0 we used aniline blue staining, with subsequent examination by UV fluorescence microscopy (Fig 1E), and we counted yellow pixels in digital images (Fig 1F). However, we did not observe any changes in callose deposition in the four mutants as compared to Col-0 plants. These observations indicated that the enhanced disease susceptibility of the four mutants was not attributable to compromised callose deposition.

It is essential for plants to maintain an intact JA-mediated signaling pathway to mount an efficient immune response towards necrotrophs. We therefore hypothesized that the enhanced susceptibility of the four TF mutants might be explained by defects in the JA pathway. We tested this hypothesis by searching for defects in induced transcript accumulation of the defense-related and JA marker *PDF1*.*2* gene, following inoculation by *P*. *cucumerina*. RT-qPCR measurements of transcript accumulation at 0, 24, 48, and 72 h post inoculation (h.p.i.) with *P*. *cucumerina* revealed that none of the mutants were compromised in JA-mediated transcriptional activation (Fig 1G). Interestingly, the JA-dependent defense response was induced to an even higher degree in the four mutants as compared to Col-0. This was particularly prominent in *bhlh99*, *zfp2* and *at1g66810* insertion mutant plants. Moreover, no differences were observed in JA-mediated root growth inhibition in Col-0 and the four mutants grown on plates containing JA, which is in contrast with the root growth observed for the JA insensitive *coi1* mutant (Fig 1H and 1I). Therefore, this excludes that the mutants are defective in a JA-mediated response triggered by the two fungal species. Similarly, *PR1* gene expression, selected as a SA-mediated defense response marker, was not compromised in the mutants and *PR1* transcript accumulation was even more prominent in the *zfp2* and *pap2* insertion mutants than in Col-0 (Fig 1G). These results, therefore, indicate that also the SA-mediated signaling is functional in the mutants.

### Transcriptomic analysis of *zfp2*, *bhlh99*, *pap2* and *at1g66810* mutant plants

**Fig 2:**
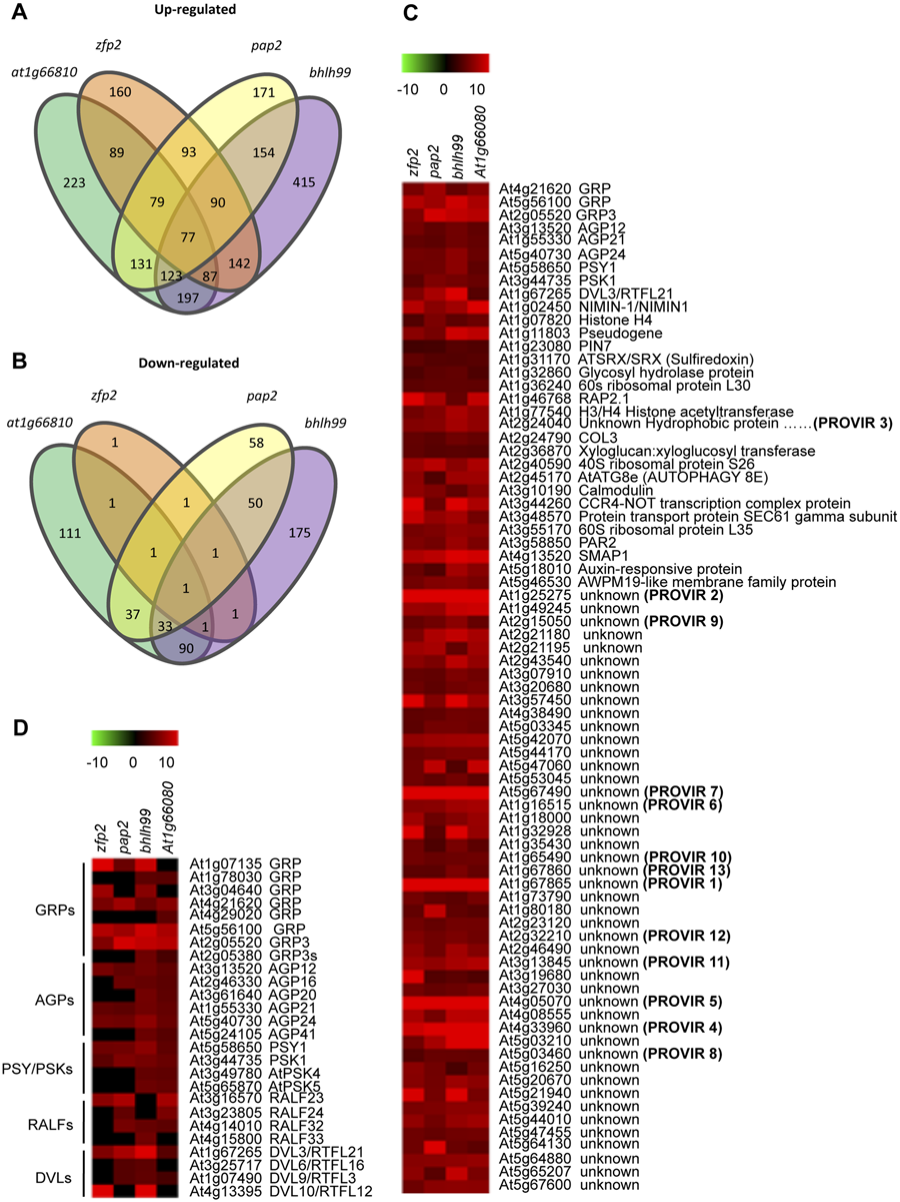
Comparative transcriptome analysis in *zfp2*, *bhlh99*, *pap2* and *at1g66810* insertion mutants. *(A-B)* Comparisons of gene expression in *zfp2*, *bhlh99*, *pap2*, and *at1g66810* insertion mutant lines depicted using Venn diagrams. Sets of genes were selected using criteria described in Material and Methods. The gene number in each mutant for each set is displayed within an ellipsoidal circle. Genes common in two, three, or four mutants are indicated in the intersection of circles, so that the sum of the numbers within a cycle for each mutant represents the total number of genes deregulated in each mutant. (**A**) Intersection of genes up-regulated in each mutant with those up-regulated in three other mutants. (**B**) Intersection of down-regulated genes in each mutant with those down-regulated in the three other mutants. (**C**) Heat map clustering of up-regulated genes commonly expressed in the four mutants. (**D**) Clustering of selected gene groups encoding other signaling peptides family members, which are different to those shown in C, but are present in at least one, two, or three mutants but not in the four mutants. The description of genes that fall into each cluster is indicated on the right. Provisionally, some genes annotated as unknown have been coined as *PROVIR1* to *PROVIR13*.

The four TF mutants showed similar enhanced disease susceptibility to necrotrophs. To further elucidate factors affecting the mutant susceptibility, we performed whole-transcriptome analysis of non-inoculated fully-expanded leaves from four-week-old plants grown under normal conditions, using two-color long-oligonucleotide microarrays. Selection of genes exhibiting at least a log2-fold change in *zfp2*, *bhlh99*, *pap2*, and *at1g66810* insertion mutant plants versus Col-0 (P < 0.05), identified differentially expressed gene sets in each of the mutants. We found a total of 183 (182 up- and one down-regulated), 735 (494 up- and 241 down-regulated), 284 (198 up- and 86 down-regulated) and 334 (255 up- and 155 down-regulated) differentially expressed genes in the *zfp2*, *bhlh99*, *pap2*, and *at1g66810* insertion mutant plants, respectively, versus Col-0 (Summarized in Fig 2A and 2B; S1–S4 Tables). We subsequently examined the number of differentially expressed genes commonly shared by the mutants, and found that 77 genes were commonly up-regulated in the four mutants (Fig 2C). *At4g18480* was the only gene commonly repressed in the four mutants, and encodes a magnesium chelatase (CHLI1), involved in chlorophyll biosynthesis. Microarray data validation was done using RT-qPCR. S2 Fig shows expression of 12 selected genes constitutively up-regulated in the four mutants, and three additional genes showing no variation in expression among the four TF mutants. The RT-qPCR results correlated well with the microarray data.

Most genes identified as commonly induced in the four TF mutants, i.e., 47 of the 77 commonly up-regulated genes, encoded proteins of unknown function. For genes annotated with a biological function, or for genes that at least showed clear homology to previously identified genes, only one was unequivocally identified as being involved in disease (i.e. NIMN-1), which negatively regulates the SA receptor NPR1 [41]. Interestingly, a substantial portion of the genes up-regulated in the four mutants encoded putative secreted peptides, which can act as local signals (peptide hormones) that mediate cell-to-cell communication during processes of cell growth, expansion, and differentiation [42]. Among these small putative hormone peptides, the bioactive five amino acid sulfated peptide growth factor PHYTOSULFOKINE (PSK) [43] and the 18 amino acid sulfated glycopeptide PLANT PEPTIDE CONTAINING SULFATED TYROSINE 1 (PSY1) [44] where notable, as they were up-regulated in all four TF mutants. Furthermore, additional tyrosine-sulfated glycopeptide isoforms, e.g., *AtPSK4* and *AtPSK5*, were up-regulated in the *bhlh99* and *at1g66810* insertion mutants (Fig 2D). Similarly, *DEVIL3/ROTUNDIFOLIA21* (*DVL3/RTFL21*), which encodes a 51 amino acid secreted polypeptide involved in the regulation of polar cell proliferation on the longitudinal axis of organs [45,46] was over-expressed in the four mutants, and three additional members of this 23 member peptide family, *DVL6/RTFL16*, *DVL9/RTFL3*, and *DVL10/RTFL12* were over-expressed in at least two or three of the four TF mutants, respectively (Fig 2D). The four TF mutants also over-expressed members of a gene family encoding RAPID ALKALINIZATION FACTOR (RALF), which are 49 amino acid secreted peptides that causes rapid apoplastic alkalinization [47,48].

Genes encoding secreted Pro/Hyp-rich ARABINOGALACTAN-PROTEINS (AGPs), in particular *AGP12* and *AGP21*, were over-expressed in the four mutants (Fig 2C), and additional AGP-related genes (e.g., *AGP16*, *AGP20*, *AGP24*, and *AGP41*) were also over-expressed in at least two of the mutants (Fig 2D). AGPs belong to a large, secreted and highly glycosylated (proteoglycans) protein family. The carbohydrate component typically constitutes 90% to 98% (w/w) of the protein and is primarily *O*-linked to the Hyp residue of the protein backbone. The carbohydrates of AGPs are usually branched type II arabino-3,6-galactans (AGs, 5–25 kD). Many Arabidopsis AGPs contain a predicted glycosylphosphatidylinositol (GPI) membrane anchor in their C-terminal domain, and mounting evidences indicate that AGPs serve a role in controlling plant growth and development via the AGPs´ anchoring specificity to lipid domains in the plasma membrane [49].

Common to the four mutants was also the over-expression of three genes encoding secreted extracellular matrix associated glycine-rich-proteins (GRPs; Fig 2C), and five additional *GRP* genes were up-regulated in the individual mutant gene sets (Fig 2D). GRPs are represented by over 30 genes in Arabidopsis [50]. The secreted GRP3 isoform is of particular interest as it interacts with the extracellular domain of the cell wall associated receptor protein kinase WAK1 [51], recently reported to mediate disease resistance in Arabidopsis [9]. This might therefore link the GRP3 protein to signal transduction events during pathogenesis.

The above described putative signaling peptides have been implicated as important regulatory molecules that coordinate cellular responses required for differentiation, growth, and stress adaptation. They are, furthermore, secreted to the extracellular matrix, where they appear to act as ligands for plasma membrane localized receptors of the same cell or in adjacent cells, which could potentiate autocrine/paracrine signaling [52,53]. Therefore, we speculate that, in the *zfp2*, *bhlh99*, *pap2* and *at1g66810* insertion mutant plants, diverse ligand-receptor signaling modules become constitutively active, which might provide a basis to explain the shared susceptibility to the fungal necrotrophs.

### The *zfp2*, *bhlh99*, *pap2* and *at1g66810* insertion mutants have altered apoplastic pH and reduced venation in cotyledons

**Fig 3:**
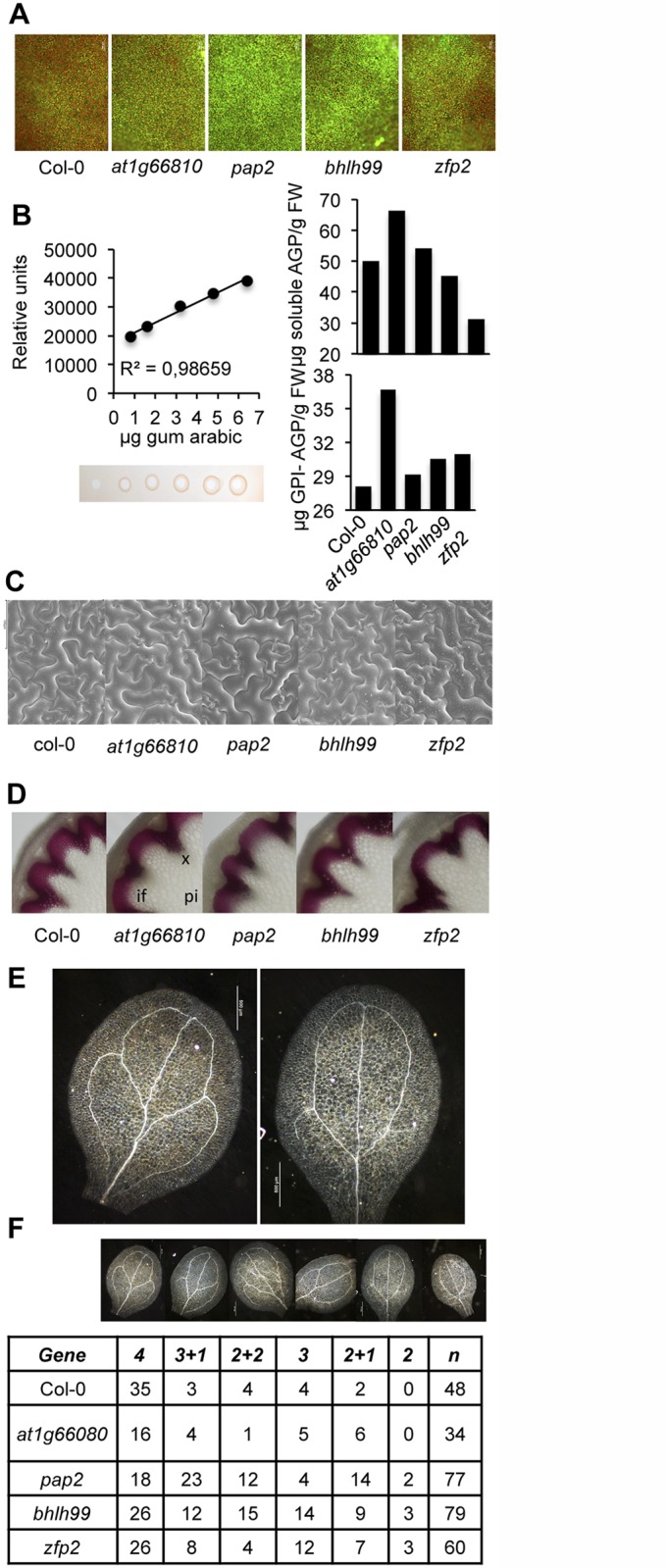
Characterization of *zfp2*, *bhlh99*, *pap2* and *at1g66810* mutants. (**A**) Oregon Green 488 dextran-derived fluorescence upon infiltration of the full expanded leaves with the apoplastic pH indicator. In comparison to Col-0, more intense fluorescence emission was observed in the four mutants, and was particularly intense in *pap2* plants, indicative of enhanced alkalinization of the apoplast in mutants compared to Col-0 plants. (**B**) Estimation of plasma membrane-anchored (GPI-AGP) and free AGP content in Col-0 and *zfp2*, *bhlh99*, *pap2*, and *at1g66810* insertion mutant plants using β-d-Glucosyl Yariv reagent. AGP content was calculated with respect to a regression curve obtained by a radial diffusion assay in agarose plates containing Yariv regent and increasing amounts of gum Arabic (calibrating curve on the left). (**C**) Scanning electron microscopy (SEM) of leaf epidermis in Col-0 and *zfp2*, *bhlh99*, *pap2*, and *at1g66810* mutants. Only *pap2* exhibited a slight increase in epidermal cell size which was variable among different leaves of different plants. (**D**) Histochemical detection of lignin in proximal stem sections of Col-0 and *zfp2*, *bhlh99*, *pap2* and *at1g66810* mutant stems. Stem sections were stained with phloroglucinol-HCl (red color) for lignin detection in the interfascicular fiber walls and xylem cells, as observed with light microscopy. if, Interfascicular fibers; pi, Pith parenchyma; x, Xylem. (**E-F**) Cotyledon vein patterns were altered in *zfp2*, *bhlh99*, *pap2*, and *at1g66810* insertion mutants. **E**, magnified pictures showing pattern defects exhibited by some mutants (e.g., 2 loops; right picture) compared to the most common 4 loop pattern observed in Col-0 (left picture). **F**, columns 2–7: number of cotyledons displaying the venation pattern depicted at the top, which ranged from the most common 4 loops observed in Col-0 to the less common phenotype of 2+1 or 2 loops, but prevalent in the *pap2*, *bhlh99*, or *zfp2* mutants. “n” = total number of scored cotyledons.

We subsequently examined whether the altered gene expression in the four mutants translated into distinct measurable cellular, physiological, and molecular phenotypes. RALF polypeptide secretion can increase apoplastic alkalinization [47,48]. Fluorescent pH sensitive dye Oregon Green 488 conjugated to a dextran molecule that prevents its movement into the symplast [54] was used to assess pH variation in the apoplast. Leaves infiltrated with this dye were imaged by fluorescent microscopy, which revealed that the four TF mutants had increased apoplastic pH compared to Col-0 plants; the *pap2* and *bhlh99* insertion mutants, in particular, showed substantial apoplastic alkalinization (Fig 3A).

To detect if the amounts of AGPs were changed in the TF mutants compared to Col-0, leaf extracts were prepared and were either treated, or not treated, with phosphatidyl-inositol-specific phospholipase C to discriminate between GPI-anchored AGPs and soluble AGPs, respectively. Extracted and solubilized AGPs were quantified by estimating AGP binding to synthetic β-glucosyl Yariv reagent in agarose plate diffusion assays; an assay calibrated using a gum arabic dilution series (Fig 3B) [55]. Our results showed that the TF mutants were enriched in GPI-AGPs, which were most conspicuous in the *at1g66810* insertion mutant, and to a minor extent in *zfp2*, *bhlh99*, and *pap2* insertion mutants (Fig 3B). Soluble AGPs, on the other hand, did not appear to over-accumulate in the mutants when compared to Col-0. Since GPI-anchored proteins in mammals and yeast are involved in cell-cell signaling, and the GPI anchor determine their location in membrane microdomains, or membrane rafts [56], we speculate that the GPI-anchored AGPs might be involved in signaling pathways, possibly together with the ligand-receptor signaling modules described above [57].

AGPs impact on plant growth and development, and similar functions have been proposed for PSY, PSKs, DVLs/RTFL, RALFs [42], and also for GRPs; the latter in particular during plant vascular development [50]. However, the four TF mutants did not exhibit alterations in gross plant morphology or in growth rates (S3 Fig). We tested if changes in expression of genes encoding the signaling peptides elicited alterations in cellular shape and coordination of cell patterning by conducting scanning electron microscopy (SEM) on leaf epidermal tissue. We did not observe any apparent differences in epidermal cells among the four mutants as compared to Col-0 using SEM (Fig 3C). Furthermore, based on phloroglucinol stained stem cross sections of the mutants and Col-0 plants, we did not observe any differences in secondary vasculature, indicating that xylem differentiation is not changed in the mutants (Fig 3D). Interestingly, the four mutants exhibited a variety of defects in cotyledon venation patterns compared to Col-0 (Fig 3E and 3F). The secondary veins in Arabidopsis cotyledons branch to form four loops without gaps (Fig 3E); however, the mutants displayed reduced venation, with fewer secondary veins in the cotyledons, and increased discontinuous venation with gaps in secondary veins (Fig 3F). The observed defects in venation patterns are reminiscent of patterns observed in certain Receptor-like Kinase (RLK) mutants, or in plants with alterated auxin and brassinosteroid hormone signaling [58], indicating that the four TF mutants could be impaired in signaling aspects or have hormone imbalances.

### Over-production of signaling peptides affect fungal disease susceptibility

**Fig 4:**
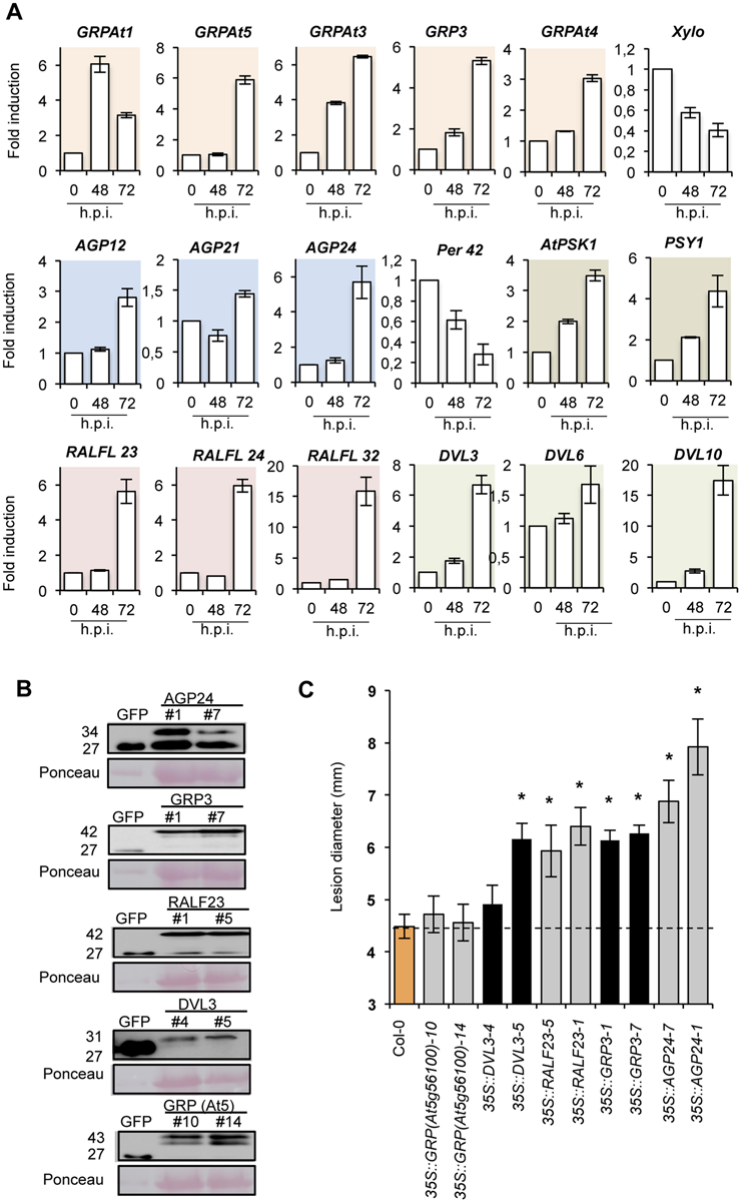
Functional implication of different signaling peptides in Col-0 disease response to *P*. *cucumerina* infection. (**A**) Expression of genes encoding distinct members of GRP, AGP, PSK1, PSY, RALF, and DVL signaling peptides in Col-0 plants at early *P*. *cucumerina* infection stages. Relative expression was assayed over a 72-h time course by quantitative RT-PCR on total RNA from leaves following inoculation with a drop of *P*. *cucumerina* spore suspension. Expression of the *Per42* peroxidase and the xyloglucan:xyloglucosyl transferase *Xylo* genes was concurrently assayed as internal controls and used for comparison. Data represent means ± SD (n = 3 biological replicates). Expression was normalized to the constitutive ACT2 gene, then to expression at each time point in mocked Col-0 plants. (**B**) Western blots with anti-GFP antibodies of crude protein extracts derived from T3 homozygous Col-0 plants expressing either a *35S*::*GFP* construct or *AGP24-GFP*, *GRP3-GFP*, *RALF23-GFP*, *DVL3-GFP* and *GRP (At5g56100)-GFP* constructs. Two independent lines for each gene construct were used and the accumulation of the encoded fusion protein compared to that of free GFP. Ponceau staining of the nitrocellulose filter confirmed equal protein loading. (**C**) Resistance response to *P*. *cucumerina* of Col-0 and two independent homozygous lines expressing each of the signaling peptides proteins fused to GFP shown in panel **B**. Disease was evaluated 11 d.p.i by determining the average lesion diameter on three leaves per plants and from 15 plants per genotype. Data points represent average lesion size ± SE of measurements. An ANOVA was conducted to assess significant differences in disease symptoms with a 0.05 level of significance. Error bars represent standard deviation (SD) (n = 12). Asterisks indicate statistical significant differences.

We argued that the up-regulated genes encoding secreted signaling peptides in the *zfp2*, *bhlh99*, *pap2*, and *at1g66810* insertion mutants were causative for the enhanced susceptibility to fungal necrotrophs. Therefore, we investigated the transcriptional response of 16 members of the *AGPs*, *RALFs*, *DVLs*, *PSK*, and *PSY* gene families identified as up-regulated in the TF mutants during a 72-h time course following inoculation of Col-0 plants with *P*. *cucumerina*. RT-qPCR analysis showed that fungal infection induced expression of most of the examined genes encoding signaling peptides (Fig 4A). Some of these genes were induced as early as 48 h.p.i (e.g., *GRPAt1*; *GRPAt3*; *PSK1*), and enhanced gene expression was observed for all of them by 72 h.p.i. These induction patterns were not always observed for the genes in the TF mutants; for example *Xylo*, encoding a xyloglucantransglycosidase, and *Per42*, encoding peroxidase 42, behaved differently in the TF mutants to that in Col-0, and were down-regulated following *P*. *cucumerina* infection (Fig 4A).

Subsequently, we examined if over-expression of some of the individual secreted signaling peptides might elicit alterations in disease susceptibility. Therefore we generated transgenic plants expressing some of the signaling peptides (e.g., AGP24, GRP3, GRP (At5g56100), RALF23, DVL3/RTFL21) fused to GFP under the control of the strong constitutive promoter *35S CaMV*. Western blot analysis using anti-GFP antibodies was used to identify and select two independent homozygous transgenic lines, which accumulated detectable amounts of the tagged proteins for each construct (Fig 4B). The response to *P*. *cucumerina* infection was subsequently characterized and compared to Col-0 plants (Fig 4C). All tagged proteins were observed in close proximity to the plasma membrane of root epidermal cells using confocal microscopy. This result contrasted with the localized expression of free GFP in the nuclei and cytosol (S4 Fig).

Transgenic lines over-expressing *GRP* (*At5g56100*) exhibited no differences as compared to Col-0 plants in disease progression induced by *P*. *cucumerina*; however, over-expression of *AGP24*, *GRP3*, *RALF23*, and *DVL3* led to enhanced disease susceptibility to the fungus (Fig 4C). The increase was comparable to, or even higher than, what we observed in the different TF mutants (Fig 1A and 1B). Disease susceptibility was most prominent in the two lines over-expressing *AGP24* (lines #1 and #7; Fig 4C). In the lines over-expressing *DVL3*, only line #5, but not line #4, showed significant enhancement in susceptibility towards *P*. *cucumerina* (Fig 4C), despite accumulation of similar levels of the DVL3-GFP fusion protein (Fig 4B), possibly indicating a negative positional effect of the T-DNA insertion for line #4. The fact that the two lines that over-accumulated GRP3-GFP and GRP(At5g56100)-GFP had contrasting effects on disease promotion and susceptibility, suggesting functional specificity for each protein. Moreover, *P*. *cucumerina* inoculation of transgenic lines over-expressing *PSY1* (e.g., lines 35S::PSY T2-11, T2-12, and T2-13), described by Amano et al. [44], also resulted in increased susceptibility to the pathogen; this enhancement was similar to that attained in *pap2* (S5 Fig).

Our results therefore suggest that many of the commonly up-regulated genes found in the four TF mutants function as positive regulators of disease susceptibility, and that their expression is induced during the course of pathogen infection.

### Identification of additional plant genes that mediate resistance to necrotrophic fungal pathogens

To assess whether also other proteins encoded by the 77 genes identified in the microarray analysis could similarly play a role in mediating disease susceptibility to *P*. *cucumerina*, we selected 13 of those annotated as encoding proteins of unknown function. The proteins were tentatively named PROVIR1 to PROVIR13 (Fig 2C). Similar to the signaling peptides described above, the encoded PROVIR factors were low molecular weight polypeptides ranging from 34 amino acids (e.g., PROVIR6) to 123 amino acids (e.g., PROVIR9) (S6 and S7 Figs). SignalP [59] identified the presence of hydrophobic N-terminal signal peptides in many PROVIRs, predicting that PROVIRs would potentially enter the secretory trafficking route. The exceptions were PROVIR6, 7, and 12, for which SignalP did not identified any signal peptides.

**Fig 5:**
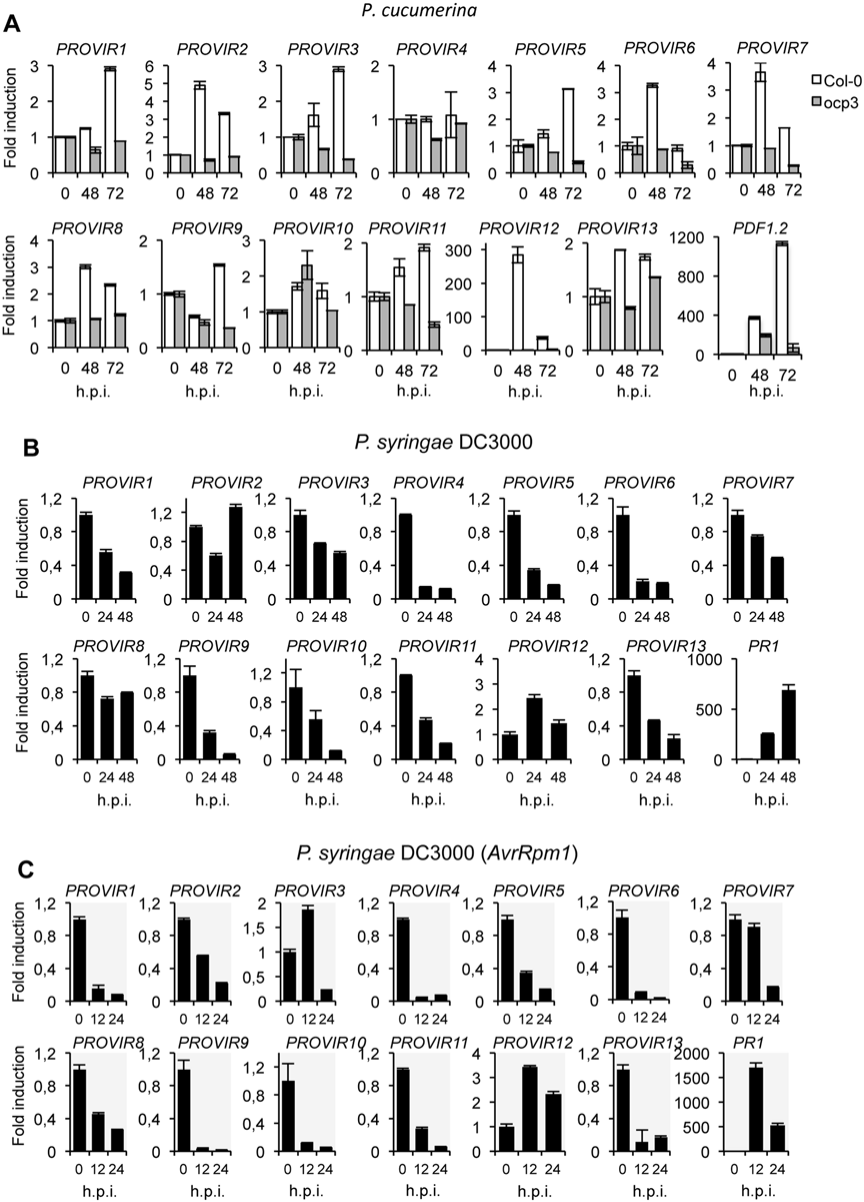
Expression patterns of *PROVIR* genes following pathogen inoculation. (**A**) *PROVIR1* to *PROVIR13* expression in Col-0 and in the disease resistant mutant *ocp3* in early *P*. *cucumerina* infection stages. Relative expression was assayed over a 72-h time course by quantitative RT-PCR on total RNA from leaves of Col-0 (white bars) and *ocp3* (grey bars) following inoculation with a drop of spore suspension of *P*. *cucumerina*. Leaf sectors centered around the inoculation point were sampled for RNA extraction. Expression of the defense-related *PDF1*.*2* gene was concurrently assayed as internal control for comparison. Data represent means ± SD (n = 3 biological replicates). Expression was normalized to the constitutive *ACT2* gene, then to expression at each time point in mocked Col-0 and *ocp3* plants. (B-C) RT-qPCR analyses showing repression of *PROVIR* gene expression upon infection with virulent *P*.*s*.DC3000 at 0, 24, and 48 post inoculation (h.p.i.) (**B**), and following infection with the avirulent *P*.*s*. DC3000 (AvrRpm1) strain at 0, 12, and 24 h.p.i. (**C**). Data represent mean ± SD, n = 3 replicates. Expression was normalized to the constitutive ACT2 gene, then to expression at each time point in mocked Col-0 plants.

With the sole exception of *PROVIR4*, all *PROVIR*s were induced during a 0-to-72 h inoculation period with *P*. *cucumerina*. The induced expression varied from two-fold (*PROVIR10*, *11* and 13) to 300-fold *(PROVIR12*) (Fig 5A). The defense-related *PDF2*.*1* marker gene was included as an internal control to verify the activation of classical defense-related host responses induced by the fungus. For some *PROVIR* genes, induction was progressive (e.g., *PROVIR1*, *3*, *5* and *11*), while for others, the gene induction was transient, e.g., peaking at 48 h.p.i., and then declining (e.g., *PROVIR2*, *6*, *7*, *8* and *12*). Interestingly, comparative expression profiling of *PROVIR* genes in *ocp3* plants, a mutant exhibiting pronounced enhancement in *P*. *cucumerina* disease resistance [17,32,33], revealed that the induction of all the *PROVIR* genes was abolished (Fig 5A). Therefore, the results suggested that fungal-induced *PROVIR* gene expression in Col-0 was congruent with lesion development and disease progression, a process that becomes obstructed in *ocp3* plants.

We also investigated *PROVIR* gene expression in Col-0 following inoculation with the biotrophic pathogen *Pseudomonas syringae* DC3000, a pathogen that requires living cells to support its growth, in contrast to the necrotrophic lifestyle of *P*. *cucumerina*. Our results demonstrated that *P*. *syringae* DC3000 infection repressed *PROVIR* gene expression. Here, *PROVIR* genes were down-regulated at 24 h.p.i to levels below those normally observed in non-inoculated healthy plants, and this repression was maintained even after 48 h.p.i. (Fig 5B). The only exception was *PROVIR12*, for which the mRNA levels were slightly induced (2-fold). In contrast, the defense-related *PR1* marker gene, used as an internal control to verify SA-mediated activation of defense-related genes diagnostic of Arabidopsis plant infection with biotrophic pathogens, was notably up-regulated. Furthermore, repression of *PROVIR* genes was even more acute when Col-0 plants were inoculated with *P*. *syringae* DC3000 carrying the avirulent *AvrRpm1* gene, a pathogenic strain that generates an incompatible plant-pathogen interaction in Col-0 plants. The repression of *PROVIR* genes was here substantial and occurred as early as 12 and 24 h.p.i. (Fig 5C). The only exception of this behavior was *PROVIR12*.

These data suggest that the PROVIR factors correlated positively with development of disease generated by a necrotrophic pathogen, but negatively with disease generated by biotrophic/hemibiotrophic pathogens in a susceptible host. Moreover, the effects were even more accentuated when the biotrophic pathogen elicited activation of programmed cell death or apoptotic cellular processes related to hypersensitive response (HR). This indicates a distinction between gene reprogramming associated with on the one hand necrosis and on the other HR.

### PROVIR factors affect disease susceptibility to *P*. *cucumerina*

**Fig 6:**
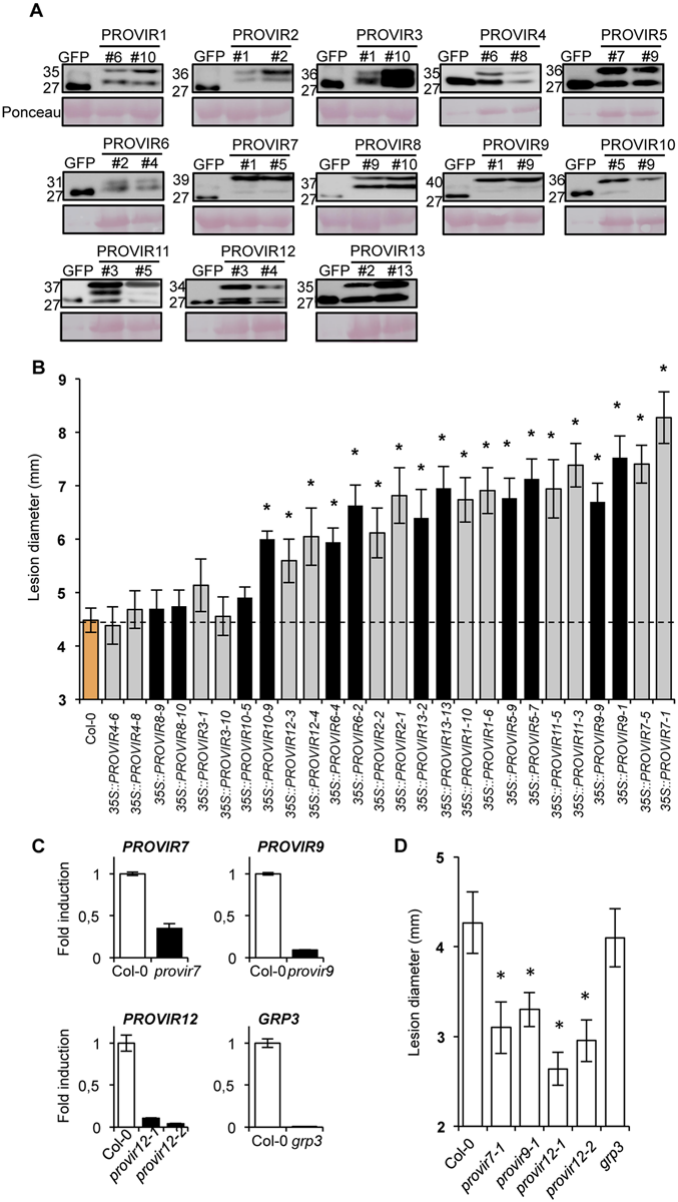
PROVIR1-to-13 overexpression confers enhanced disease susceptibility to *P*. *cucumerina* infection. (**A**) Western blots with anti-GFP antibodies of crude protein extracts derived from T3 homozygous Col-0 plants expressing either a 35S::GFP construct or the respective PROVIR1-to-13::GFP constructs. Two independent lines for each PROVIR::GFP construct were used and the accumulation of the encoded fusion protein was compared to that of free GFP. Equal protein loading was check by Ponceau staining of the nitrocellulose filter. (**B**) Resistance response to *P*. *cucumerina* of Col-0 and two independent homozygous lines expressing each of the respective PROVIR::GFP proteins shown in panel **A**. Disease was evaluated 11 d.p.i. by determining the average lesion diameter on three leaves per plant from 15 plants per genotype. Data points represent average lesion size ± SE of measurements. An ANOVA was conducted to assess significant differences in disease symptoms, with a priori *P* < 0.05 level of significance; significant differences are indicated with letters. (**C**) Accumulation levels of endogenous *PROVIR7*, *PROVIR9*, *PROVIR12*, and *GRP3* transcripts measured in comparatively healthy Col-0 plants (left bars) and in T-DNA *provir7*, *provir9*, *provir12-1*, *provir12-2*, and *grp3* T-DNA insertion mutants (right black bars). Data represent mean ± SD; n = 3 biological replicates. Expression was normalized to the constitutive *ACT2* gene, then to expression in Col-0 plants. (**D**) Resistance response to *P*. *cucumerina* of Col-0 and *provir7*, *provir9*, *provir12-1*, *provir12-2*, and *grp3* plants. Disease was evaluated as in **B**.

Similar to the secreted signaling peptides, we investigated whether over-expression of the PROVIR factors could alter disease susceptibility to the necrotroph *P*. *cucumerina*. Western blot analysis with anti-GFP antibodies was again used to select two independent homozygous transgenic lines accumulating detectable amounts of the corresponding PROVIR-GFP fusion proteins (Fig 6A). The response of each individual transgenic line to *P*. *cucumerina* infection was then characterized (Fig 6B). PROVIR over-production did not affect growth or development of the transgenic plants (S8 Fig). Disease progression did not appear different in transgenic lines that over-accumulated PROVIR3, 4, or 8 as compared to Col-0 plants (Fig 6B). In contrast, plants that over-accumulated PROVIR6 or 12 showed significant increase in the formation of necrotic lesions induced by the fungi. *PROVIR10* over-expression did also lead to enhanced susceptibility to the fungi, determined by increased lesion diameter; however, this observation was only evident in one of the two transgenic lines (i.e., line #9) despite that the other line (i.e., line #5) contained higher amounts of the PROVIR10-GFP protein (Fig 6A). Moreover, enhanced disease susceptibility was prominent in the remaining transgenic lines, i.e. those plants that over-accumulated PROVIR1, 2, 5, 7, 9, 11, or 13 (Fig 6B). Therefore, over-production of the majority of the selected PROVIR factors resulted in *P*. *cucumerina* disease susceptibility. These results, together with the induction of the PROVIR factors by fungal inoculation, argue in favor of a mechanism where the signaling peptides work in concert as pro-virulent or disease susceptibility factors for fungal necrotrophs.

As a complement to these studies, we searched for T-DNA insertion mutants defective in the PROVIRs. However, only a few T-DNA mutants were available via the stock center, presumably due to the small sizes of the genes. However, we did find T-DNA insertion lines corresponding to *PROVIR7*, *PROVIR9*, *PROVIR12*, and *GRP3*. Furthermore, these lines showed reduced expression levels of the corresponding genes (Fig 6C, S9 Fig). *P*. *cucumerina* infection assays revealed that *provir7*, *provir9*, and the two allelic mutants *provir12-1* and *provir12-2*, were more resistant to fungal disease (Fig 6D), a phenotype congruent with the characterization of *PROVIR7*, *9*, and *12* over-expression lines (Fig 6B). Despite very low *GRP3* mRNA levels in the *grp3* insertion mutant line, *P*. *cucumerina* disease susceptibility remained unaltered in this line and was comparable to that of Col-0. The absence of phenotype might be due to redundant functions of related genes since the *GRP* gene family is large in Arabidopsis [50].

### PROVIR factors are mainly localized to the plasma membrane and periplasm

**Fig 7:**
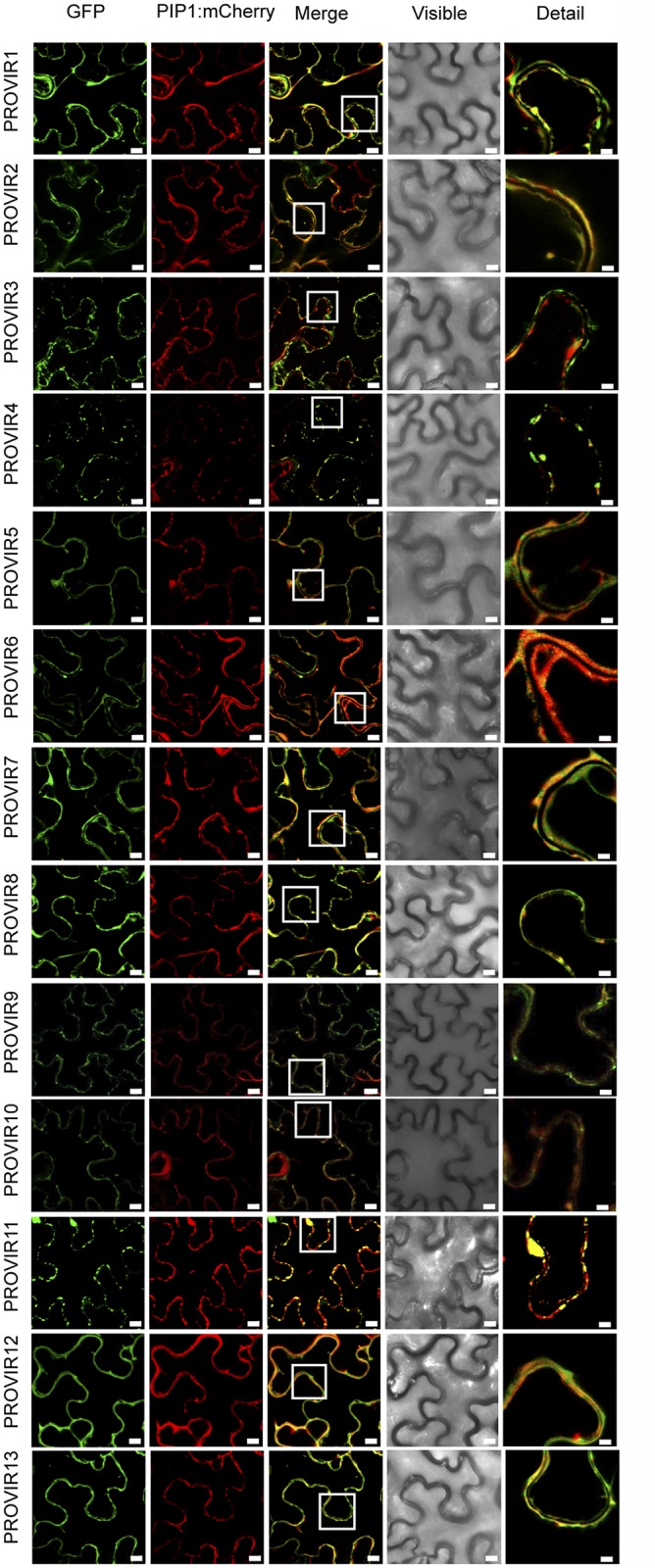
Localization of different PROVIR-GFP proteins in *N*. *benthamiana* leaves and protein co-localization with the plasma membrane marker protein PIP-mCherry by confocal microscopy. Expression of PROVIR1-to-13-GFP (72 h.p.i.) resulted in distinct pericellular fluorescence patterns distributed either linearly along the plasma membrane or forming punctuated foci resembling small membrane clusters. Co-expression of PROVIR-GFP with the plasma membrane marker PIP-mCherry facilitated tracing the plasma membrane. Scale bars are 8 μm, except for the right longitudinal panel where scale bars are 2 μm. This provides a magnified detail of the boxed sector in the intermediate panel merging GFP and mCherry-derived fluorescence.

As indicate above, most of the PROVIR factors carry a signal peptide required for translocation along the endomembrane system (S6 and S7 Figs). PROVIR subcellular localization was determined by fusing GFP to the C-terminus of the full length PROVIR sequence. The constructs, driven by a 35S promoter, were expressed in *Nicotiana benthamiana* leaves using agro-infiltration. Fusion protein localization was assessed by confocal microscopy. The same gene constructs were used to generate the transgenic Arabidopsis plants indicated above. All PROVIR-GFP fusions expressed in *N*. *benthamiana* showed fluorescent signals predominantly localized to the cell periphery, presumably in association with the PM (Fig 7). PROVIR pericellular and PM associations were also observed in stable transgenic Arabidopsis plants (e.g., in root epidermal cells) expressing individual *35S*::*PROVIR-GFP* gene constructs (S10 Fig), and similar localizations were also in Arabidopsis expressing AGP24-GFP, GRP3-GFP, RALF23-GFP, and DVL3-GFP (S4 Fig).

Each GFP-tagged PROVIR polypeptide was, furthermore, co-expressed with a construct bearing the plasma membrane integral protein PIP1 fused to monomeric cherry fluorescent protein (mCherry) in *N*. *benthamiana* leaves, which is a plasma membrane marker (Fig 7). PROVIR1, 2, 5, 6, 7, 8, 10, 12, and 13 fluorescent signals coincided with the PIP1-mCherry signal. PROVIR3, and 4, and to a lesser extent PROVIR9 and 11, showed some coincidence with PIP, but only at discrete sites. Expression of these latter four PROVIR factors resulted in labeling of large membrane domains, which might resemble endomembrane structures.

**Fig 8:**
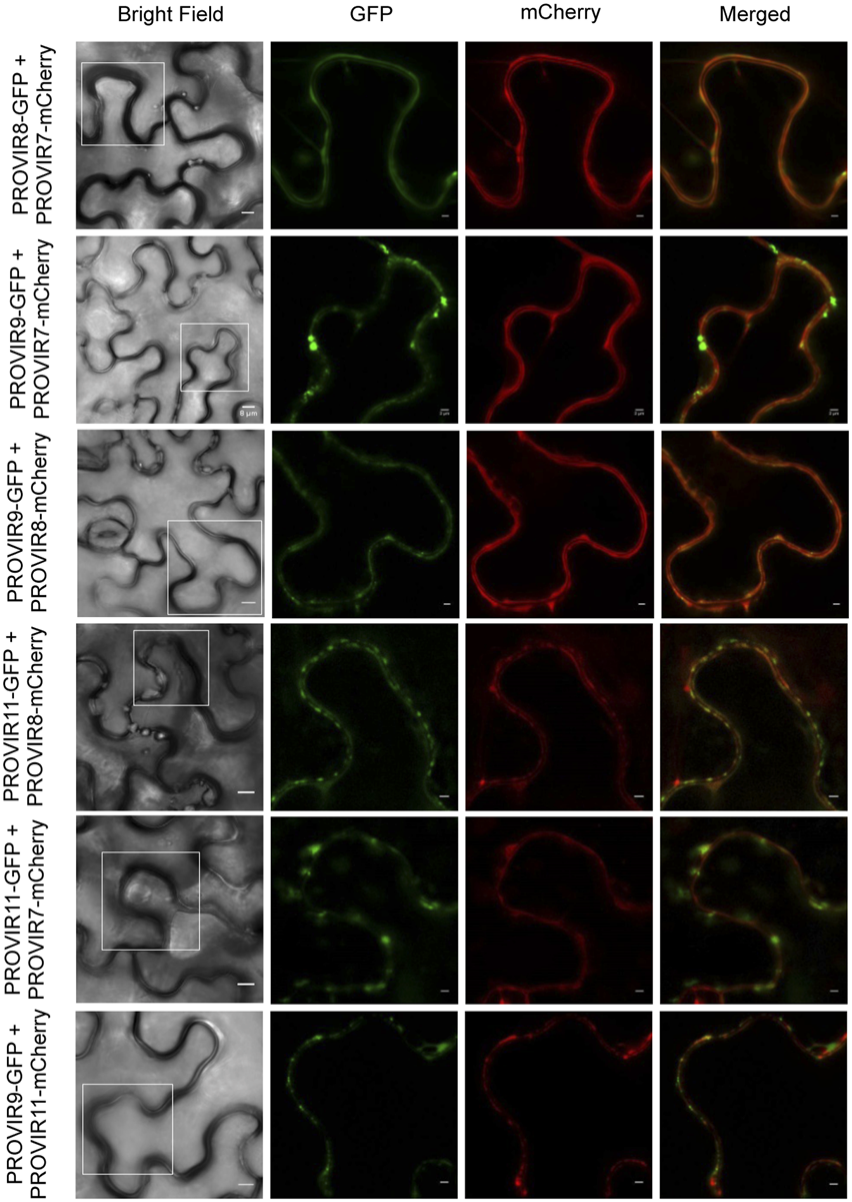
Co-localization of PROVIR proteins in *N*. *benthamiana* leaves. Co-expression of PROVIR 8, 9, and 11 proteins tagged to GFP with respective PROVIR 7, 8, and 11 proteins tagged to mCherry allowed identification of co-localization sites for PROVIR factors along or at discrete plasma membrane domains. Bright field left panel: scale bars are 8 μm. Fluorescent panels: scale bars are 2 μm, which provides a magnified detail of the boxed sectors in the left panel.

Co-expression of selected PROVIR factors fused to GFP, or to mCherry, revealed that several of the different PROVIR proteins (e.g., PROVIR7 *vs*. PROVIR8; PROVIR7 *vs*. PROVIR9; PROVIR7 *vs*. PROVIR 11; PROVIR8 *vs*. PROVIR9; PROVI8 *vs*. PROVIR11; and PROVIR9 *vs*. PROVIR11) (Fig 8) co-localized at the PM, or at discrete subdomains of this compartment.

## Discussion

Disease susceptibility caused by infectious pathogens affects most plants in their natural environment, and plants combat the majority of intruders by activation of elaborate immune responses, which typically results in an effective disease resistance response. Nevertheless, in many cases, pathogen adaptation provides a bypass to a plant’s defense and susceptibility to attackers appears. However, other molecular mechanisms serve as a foundation for immune-response independent processes in plant cells, which allow further ingress of the invading pathogen, and contribute to plant susceptibility to pathogens [60].

In this study, we identified TFs that are co-regulated with *MYB46* and *CESA4*, *7*, and *8*, that are involved in disease susceptibility to fungal necrotrophs [21,24]. Our rationale was that genes co-regulated with *MYB46* and *CESA4/7/8* may be involved in related biological processes and therefore also regulate critical aspects of disease resistance/susceptibility to necrotrophs. We found that several TFs, in particular ZFP2, BHLH99, PAP2, and AT1G66810, are required for plant resistance to fungal necrotrophs. Arabidopsis mutants defective in any of these four TFs showed remarkable disease susceptibility to *B*. *cinerea* and *P*. *cucumerina*. Interestingly, the disease susceptibility of the four mutants was not due to defects in immune signaling, since JA- and SA-responsive genes, which are the two main defense-signaling pathways in Arabidopsis, were not compromised. Callose deposition, thought to function as the first-line defense barrier to these fungal pathogens [17], was also not compromised in the mutants. These observations indicate that other host processes that contribute to plant disease susceptibility were activated in the mutants. The identified TFs might somehow be part of a “late damage control” machinery of the plant rather than the “detect and destroy” mechanism that traditionally marks the front-line immune systems.

Whole genome transcriptome analysis of Col-0, *zfp2*, *bhlh99*, *pap2* and *at1g66810* insertion mutant plants revealed that the four mutants shared 77 up-regulated genes. A substantial proportion of these genes encoded secreted peptides, which might act as local signals (peptide hormones) mediating cell-to-cell communication reported to function in the plant-specific paracrine/autocrine system [42,52,53]. These results also indicate that the four TFs redundantly activate similar signal molecules and/or pathways to fulfill related cellular purposes.

Peptide receptors are typically part of the receptor-like kinase (RLK) protein family, which may recognize different types of signaling peptides. Several of these receptors are leucine rich repeat (LRR)-RLKs or receptor-like proteins (LRR-RLPs), which can oligomerize upon peptide binding, and subsequently relay messages via phosphorylation cascades [61]. The expression of the signaling peptide family members was induced in Col-0 plants following *P*. *cucumerina* inoculation, and over-expression of the signaling peptides led to enhanced *P*. *cucumerina* susceptibility. We speculate that the production and recognition of specific ligand peptides might elicit signaling processes that offer certain fungal advantages, either by improving the recognition of, or the attachment to, the host, or simply by improving the development of the pathogen, which could contribute to the enhanced susceptibility. Thus, some pathogen species might have adapted to these plant processes to improve the invasion of the host. These non-immune related processes in the plant, therefore, contribute to the susceptibility to pathogens.

In addition to the signaling peptides, we further characterized thirteen genes from the common transcriptional signatures of the hyper-susceptible *zfp2*, *bhlh99*, *pap2* and *at1g66810* mutants. These genes encode proteins of unknown function, and were here coined as *PROVIR1 to 13*. Over-expression of many of the *PROVIR* factors in Arabidopsis conferred increased susceptibility to the necrotroph *P*. *cucumerina*, and, conversely, loss-of-function mutants conferred increased resistance. These observations indicate that most PROVIR factors were important for fungal pathogenesis. Although the functions of the PROVIR proteins remain to be elucidated, the common PROVIR association with the PM/apoplast, suggest that the PROVIR polypeptides may work in signaling processes via membrane receptors, or perhaps as scaffolding proteins in larger protein complexes as they are lacking clear GPI-anchoring and transmembrane domains. However, post-translational modification, such as palmitoylation cannot be excluded. Palmitoylation alone is typically not sufficient to anchor proteins to the PM, but might serve as an auxiliary system. Moreover, PROVIR factors were easily solubilized with neutral extraction buffers lacking detergents, suggesting that their membrane association is weak, presumably through protein-protein or ionic interactions. Understanding the mechanism of how PROVIR proteins specifically localize to membranes is, however, a challenge for future research. Nevertheless, the PROVIR localization resembles other membrane-associated proteins of unknown function, which were recently described as remorin proteins during microbe and plant symbiosis [62].

Our findings provide a novel principle of plant host disease susceptibility to fungal necrotrophs; a process where knowledge is limited when compared with the large number of molecules and proteins identified to mediate susceptibility to biotrophic and hemibiotrophic plant pathogens [60]. Characterization of the remaining factors of the common 77 member that showed transcriptional changes in the TF mutants, as well as deeper functional characterization of the PROVIR1-13 factors and the additional signaling peptides identified here, should allow us to uncover molecular processes that underlie immune-response independent aspects of plant disease susceptibility. This course of study can provide new ways to develop strategies in breeding programs aimed at increasing crop disease resistance.

## Materials and Methods

### Plants growth conditions

Arabidopsis thaliana plants were grown in a growth chamber (19–23°C, 85% relative humidity, 100 mEm22 sec21 fluorescent illumination) on a 10-hr-light and 14-hr-dark cycle as previously described [63]. All mutants are in Col-0 background.

### Gene constructs and transgenic lines

For the different GFP and-mCherry constructs, each of the corresponding full length cDNA for the selected signaling peptides or PROVIR factors was amplified by PCR using Pfu DNA polymerase (Stratagene, San Diego, CA) and specific primers including Gateway adapters, and recombined into pDONR221/207 using BP ClonaseMixII kit (Invitrogen). After sequencing, all constructs were recombined into pEarleyGate101 and pB7FWG2 destination vector using LR ClonaseMixII kit (Invitrogen) and introduced into Col-0 plants via Agrobacterium transformation. Cloning of the different ORFs employed in the present work and their fusion with the indicated fluorescent tag was done in a similar way. List of primers used for cloning purposes is provided in Supplementary information.

### Transient expression in *Nicotiana benthamiana* leaves

Almost fully expanded leaves were infiltrated with a suspension of *Agrobacterium tumefaciens* C58 bearing the relevant construct in 10 mM MES pH 5.6, 10 mM MgCl2, 150 mM acetosyringone at an OD600 = 0.5. After 3 days, fluorescence was analyzed in infiltrated leaves by confocal microscopy. For co-infiltration, Agrobacterium cultures grown separately and processed as indicated above, were adjusted to an O.D. = 0.5, and mixed prior to infiltration. *Agrobacterium* expressing the viral silencing suppressor P19 was included in all infiltrations.

### Confocal laser-scanning microscopy

Plant tissue was observed with a Leica TCS LS spectral confocal microscope using and HCX PL APO 640/1.25–0.75 oil CS objective. GFP-derived fluorescence was monitored by excitation with 488- and 514-nm argon laser lines, respectively, and emission was visualized with a 30-nm-width band-pass window centered at 515 nm. When GFP and mCherry were used, excitation was performed by means of a 543-nm green-neon laser line, and fluorescence emission was collected at 695 to 630 nm.

### RNA extraction, RT, and qPCR

Total RNA was extracted using TRIzol reagent (Invitrogen) following the manufacturer’s recommendations and further purified by lithium chloride precipitation. For reverse transcription, the RevertAid H Minus First Strand cDNA Synthesis Kit (Fermentas Life Sciences) was used. Quantitative PCR (qPCR) amplifications and measurements were performed using an ABI PRISM 7000 sequence detection system, and SYBR-Green (Perkin-Elmer Applied Biosystems). ACTIN2/8 was chosen as the reference gene. Primers for amplicons covering each of the genes studied are listed below in Supplemental information.

### Western blots

Protein crude extracts were prepared by homogenizing ground frozen leaf material with Tris-buffered saline (TBS) supplemented with 5 mM DTT, protease inhibitor cocktail (Sigma-Aldrich). Protein concentration was measured using Bradford reagent; unless otherwise indicated 20 μg of total protein was separated by SDS-PAGE (12% acrylamide w/v) and transferred to nitrocellulose membranes. The membranes were stained with Ponceau-S after transfer, and used as a loading control. Unless otherwise indicated, immunoblots were incubated with the indicated primary antibodies at the appropriate dilution and developed by chemiluminescence using an anti-IgG peroxidase antibody (Roche) at a 1:1000 dilution and Western Lighting plus-ECL substrate (Perkin-Elmer).

### *Botrytis cinerea* and *Plectosphaerella cucumerina* bioassays

In both *B*. *cinerea* and *P*. *cucumerina* infections, five-week-old plants were inoculated as described [32,33], with a suspension of fungal spores of 2.5x10^4^ and 5x10^6^ spores/mL respectively. The challenged plants were maintained at 100% relative humidity. Disease symptoms were evaluated by determining the lesion diameter of at least 50 lesions 11 days after inoculation. For pathogen-induced callose deposition analyses, infected leaves were stained with aniline blue and callose deposition quantifications were performed as described by Garcia-Andrade et al. [17].

### *P*.*Syrinage* DC3000 inoculations

Arabidopsis leaves were infected with *P*.*syringae* DC3000, carrying or not AvrRpm1, as previously described [64].

### T-DNA Arabidopsis mutants

Homozygous lines of T-DNA insertion mutants were identified by PCR using primers listed in Supplemental information.

### Microarray analysis

RNA obtained from leaves was amplified with the MessageAmp aRNA amplification kit from Ambion (www.ambion.com) following the instruction manual. To allow later labelling with Cy fluorophores, aminoallyl UTP was added to the mix of the T7 RNA polymerase-driven aRNA amplification reaction. The amount and quality of aRNA obtained were assessed as previously described [23]. The aminoallyl-labelled aRNA (10 mg) was incubated in 1 M Na_2_CO_3_ with 8 nmol of dye monofunctional NHS ester (Cy3/Cy5) RPN 5661 (Amersham Biosciences; www.gehealthcare.com) at room temperature in the dark for 1 h. Then, 35 L of 0.1 M sodium acetate, pH 5.2, was added and incubated for a further 5 min in the dark. The Cy-labelled aRNA was purified with the Megaclear kit from Ambion and measured with the Nanodrop ND-100 spectrophotometer. Three biological replicates were independently hybridized for transcriptomic comparison using Arabidopsis (V4) 4x44K Gene Expression Microarrays (G2519F, Agilent Technologies) that contain 43,803 oligonucleotide probes (60-mer) corresponding to 29,030 different genes (virtually the complete transcriptome) as described [23].

Preparation of Microarray slides, hybridization conditions, scanning and normalization and log transformation were previously described [23]. The mean of the three replicate log-ratio intensities and their SD values were generated. The expression data were normalized and statistically analyzed using the RankProd package in Bioconductor [65,66]. Lists of up- or down-regulated genes were selected based on the estimated percentage of false positive predictions (pfp), which is also known as false discovery rate (FDR), according to Hong *et al*. [66]. The expected FDR was controlled to be less than 5%. Genes were considered to be differentially expressed if the corrected P values (or q values) were less than 0.05. In addition, only genes with more than 2-fold change were considered for further analysis.

Microarray analyses were carried out with RNA derived from Col-0 plants and from *zfp2*, *bhlh99*, *pap2*, and *at1g66810* mutant plants. Following the above criteria, genes exhibiting altered expression in each mutant plant versus Col-0 0 were identified. Then, genes up-regulated in the individual mutants were compared to identify those genes that were commonly up-regulated in the four mutant backgrounds. These commonly up-regulated genes were selected for further functional analysis.

### Oregon Green 488 staining

A volume of 50 μL of a 25 μM Oregon Green 488 dextran (Invitrogen GmbH, Darmstadt, Germany) dissolved in deionized water was infiltrated into the leaf apoplast of intact Arabidopsis leaves with the aid of a syringe without needle, by pressing carefully onto the abaxial leaf side. The loaded area appeared darker than its surroundings. Images were taken 1h after the infiltrations as described in Geilfus and Mühling [54].

### Detection of Arabinogalactan proteins

Arabinogalactan proteins were extracted as described in Popper [55] with minor modifications. Briefly, 10 g of plant material was grinded and 10 mL of extraction buffer (50 mM Tris–HCl, pH 8, 10 mM EDTA, 0.1% v/v β-mercaptoethanol, 1% w/v TritonX-100) was added. The extract was incubated at 4°C for at least 3 h. Then, it was centrifuged for 10 min at 4,000 × *g* and the supernatant was removed. Polysaccharides and glycoproteins were precipitated with 5 volumes of ethanol at 4°C for at least 16 h. Samples were centrifuged 2 min at 2,000 × *g* and the pellet was resuspended in 5 mL of 50 mM Tris–HCl, pH 8. The suspensions were centrifuged at 10 min at 4,000 × *g* and the supernatant was collected into a polypropylene tube. The remaining pellet was resuspended in 5 mL 50 mM Tris–HCl, pH 8 and centrifuged for 10 min at 4,000 × *g*. The supernatant was removed, pooled it with that collected before, frozen and freeze dried. Finally, the dried supernatant was dissolved in 500 μL 1% w/v NaCl.

For the GPI anchored AGPs extraction, crude extracts were treated, as described in Lisanti et al. [67], with 8 units/mL of PI-PLC (Phospholipase C Phosphatidylinositol-specific; Sigma-Aldrich, Madrid, Spain) in 20 mM Hepes-buffered saline pH 7.3, containing 0.1 mM CaCl_2_ and 0.1 mM MgCl_2_) for 1h at room temperature.

The arabinogalactan proteins were detected using an agarose gel containing β-d glucosyl Yariv reagent as described [55]. A core borer was used to cut out wells in the gel containing the Yariv reagent (Biosuppliers Australia Pty Ltd., Victoria, Australia). 20–50 μL of 1% w/v NaCl was loaded in one well and into other wells a dilution series of gum arabic starting with 0.8 to 6.4 mg/mL were loaded. 5 μL the extract prepared as described before were loaded into the remaining wells, using 3 wells per sample. Plates were left in the dark 48h and pictures of the diffusion area were taken. The amount of arabinogalactan proteins per sample was calculated based in the diameter of the gum arabic dilutions.

### Scanning electron microscopy

Pictures of 2 week-old plants grown *in vitro* in MS media were taken with a JSM-5410 scanning electron microscope (SEM) (JEOL, Tokyo, Japan) in the Electron Microscopy Service of Universidad Politécnica de Valencia, Spain.

### Vein pattern characterization

Two-week-old seedlings were fixed and cleared as described [58]. Upon embedding for 1 hr in acetic acid:95% ethanol (1:3), cotyledons cleared sequentially in 70% ethanol for 30 min, 100% ethanol overnight, and 10% NaOH for 1 hr at 42C. Leaf samples were mounted on slides in 50% glycerol and observed under dark field illumination or in differential interference contrast mode to view lignified tracheary elements of the xylem.

The microarrays data have been submitted to the Gene Expression Omnibus databases under accession number GSE36308 (GSM886777-886779 for *At1g66810* mutant; GSM886780-886782 for *pap2* mutant; GSM886783-886785 for *bhlh99* mutant and GSM886786-886788 for *zfp2* mutants).

### Accession numbers

At1g66810, At4g29080, At5g65320, At5g57520, At3g61910, At5g25830, At3g49930, At1g62360, At4g01680, At1g17950, At2g38090, At5g16600, At5g62380, At1g12260, At2g44745, At4g39410, At1g74660, At5g18090, At5g03460, At4g33960, At4g05070, At3g13845, At2g32210, At1g67865, At1g67860, At1g65490, At1g16515, At5g67490, At2g15050, At1g25275, At4g21620, At5g56100, At2g05520, At3g13520, At1g55330, At5g40730, At5g58650, At3g44735, At1g67265, At2g24040, At3g49780, At5g65870, At3g16570, At3g23805, At4g14010, At4g15800, At3g25717, At1g07490, At4g13395, At5g24105, At3g61640, At2g46330, At3g13520

## Supporting Information

S1 Fig Coexpression gene vicinity network around the *MYB46* node. Nodes indicate individual genes, and edges indicate whether two genes are co-expressed above a certain mutual rank. The color edges indicate strength of the co-expression based on mutual rank relationships between the individual gene pairs. Green, orange, and red edges indicate a mutual rank relationship ≤10 (green), between 11 and 20 (orange) and 21 and 30 (red), respectively, for each connected gene. MYB46 and CESA4, CESA 7 and CESA8 encoding genes are highlighted and boxed in blue. The identified and co-regulated MYB46, VND6, At1g66810, VND4, and NAC073 transcription factor encoding genes are boxed in black. The network was generated, and modified from AraGenNet (http://aranet.mpimp-golm.mpg.de/aranet; Mutwil et al., 2010).
(TIF)

S2 Fig Validation of microarrays. Expression levels of selected genes in Col-0 plants in comparison to *at1g66810*, *pap2*, *bhlh99* and *zfp2* mutants. Relative expression was assayed by quantitative RT-PCR on total mRNA isolated from leaves. Data represent mean ± SD (n = 3 biological replicates). Expression was normalized to the constitutive ACT2 gene.
(TIF)

S3 Fig Normal development and appearance of *at1g66810*, *pap2*, *bhlh99*, and *zfp2* mutants in comparison to the Col-0 parental line. Plants were grown as indicated in Materials and Methods and pictures were taken at the vegetative stage of growth at 25 days after sowing.
(TIF)

S4 Fig Localization of free GFP and AGP24-GFP, GRP3-GFP, GRP(At5g56100)-GFP, RALF23-GFP, DVL3-GFP in transgenic Arabidopsis by confocal microscopy. Left panel shows GFP localizations in epidermal cells of roots of transgenic plants expressing each fusion protein. Right panel shows a magnification of the tissue section shown in the left panel. Observe the intense nuclear localization of free GFP compare to the pericellular localization for each of the fusion proteins.
(TIF)

S5 Fig Disease resistance response of three *35S*::*PSY* overexpressing lines to *P*. *cucumerina* and comparison to that of Col-0 and *pap2* plants. Lesion diameter of 20 plants per genotype and four leaves per plant were determined 11 d following inoculation with *P*. *cucumerina*. Values are means and ± SE (n = 80). ANOVA detected significant differences at the *P* < 0.05 level.
(TIF)

S6 Fig Deduced amino acid sequence and hydropathy plots of PROVIR1 to PROVIR7 polipeptides characterized in the present study. The theoretical molecular weight (Da) of the encoded protein, the length of the pre-protein, of its signal peptide (SP) and of the mature protein, in amino acids, is indicated in parenthesis. The amino acid sequence of the SP is indicated in bold. The hydropathy plot is indicated on the right of each amino acid sequence.
(TIF)

S7 Fig Deduced amino acid sequence and hydropathy plots of PROVIR8 to PROVIR13 polipeptides characterized in the present study. The theoretical molecular weight (Da) of the encoded protein, the length of the pre-protein, of its signal peptide (SP) and of the mature protein, in amino acids, is indicated in parenthesis. The amino acid sequence of the SP is indicated in bold. The hydropathy plot is indicated on the right of each amino acid sequence.
(TIF)

S8 Fig Normal development and appearance of PROVIR1 to PROVIR13 overexpressing transgenic strains in comparison to Col-0 wild-type plants. Plants were grown as indicated in Materials and Methods and pictures were taken at the vegetative stage of growth at 28 days after sowing.
(TIF)

S9 Fig *provir7* (At5g67490), *provir9* (At2g15050), *provir12-1* (At2g32210), *provir2-2* (At2g32210) and *grp* (At2g05520) T-DNA insertion mutants. The *provir7* mutant (strain GK-857H02) carries a T-DNA insertion internal to the unique exon and therefore disrupts the ORF. The *provir9* mutant (strain SALK-139292) carries a T-DNA insertion upstream and proximal of the ATG initiation codon and therefore could affect expression of the gene. The *provir12*-1 mutant (strain SALK-077303) carries a T-DNA insertion upstream and proximal of the ATG initiation codon and therefore could affect expression of the gene. The *provir12-2* mutant (strain SALK-077303) carries a T-DNA insertion internal to the second intron and therefore could alter mRNA stability and affect expression of the gene. The *grp3* mutant (strain SALK-012941) carries a T-DNA insertion upstream and proximal of the ATG initiation codon and therefore could affect expression of the gene. Exons are indicated with solid rectangles. T-DNA insertions are indicated with white rectangles.
(TIF)

S10 Fig Localization of free GFP and PROVIR1-GFP to PROVIR13-GFP fusions in transgenic Arabidopsis by confocal microscopy. Left panel shows GFP localizations in epidermal cells of roots of transgenic plants expressing each fusion protein. Right panel shows a magnification of the tissue section shown in the left panel. Observe the intense nuclear localization of free GFP compare to the pericellular localization for each of the fusion proteins.
(TIF)

S1 Table Genes up- and down-regulated (≥2 fold) in the Arabidopsis *At1g66810* mutant with respect to Col-0 wild type plants. (XLSX)

S2 Table Genes up- and down-regulated (≥2 fold) in the Arabidopsis *bhlh99* mutant with respect to Col-0 wild type plants. (XLSX)

S3 Table Genes up- and down-regulated (≥2 fold) in the Arabidopsis *pap2* mutant with respect to Col-0 wild type plants. (XLSX)

S4 Table Genes up- and down-regulated (≥2 fold) in the Arabidopsis *zfp2* mutant with respect to Col-0 wild type plants. (XLSX)

S1 Text Primer sequences. (DOCX)
