# Supplementary figures and images for: Novel Disease Susceptibility Factors for Fungal Necrotrophic Pathogens in Arabidopsis
Source: PLoS Pathog. 2015 Apr 1;11(4):e1004800. doi: 10.1371/journal.ppat.1004800 (PMC4382300; doi:10.1371/journal.ppat.1004800)

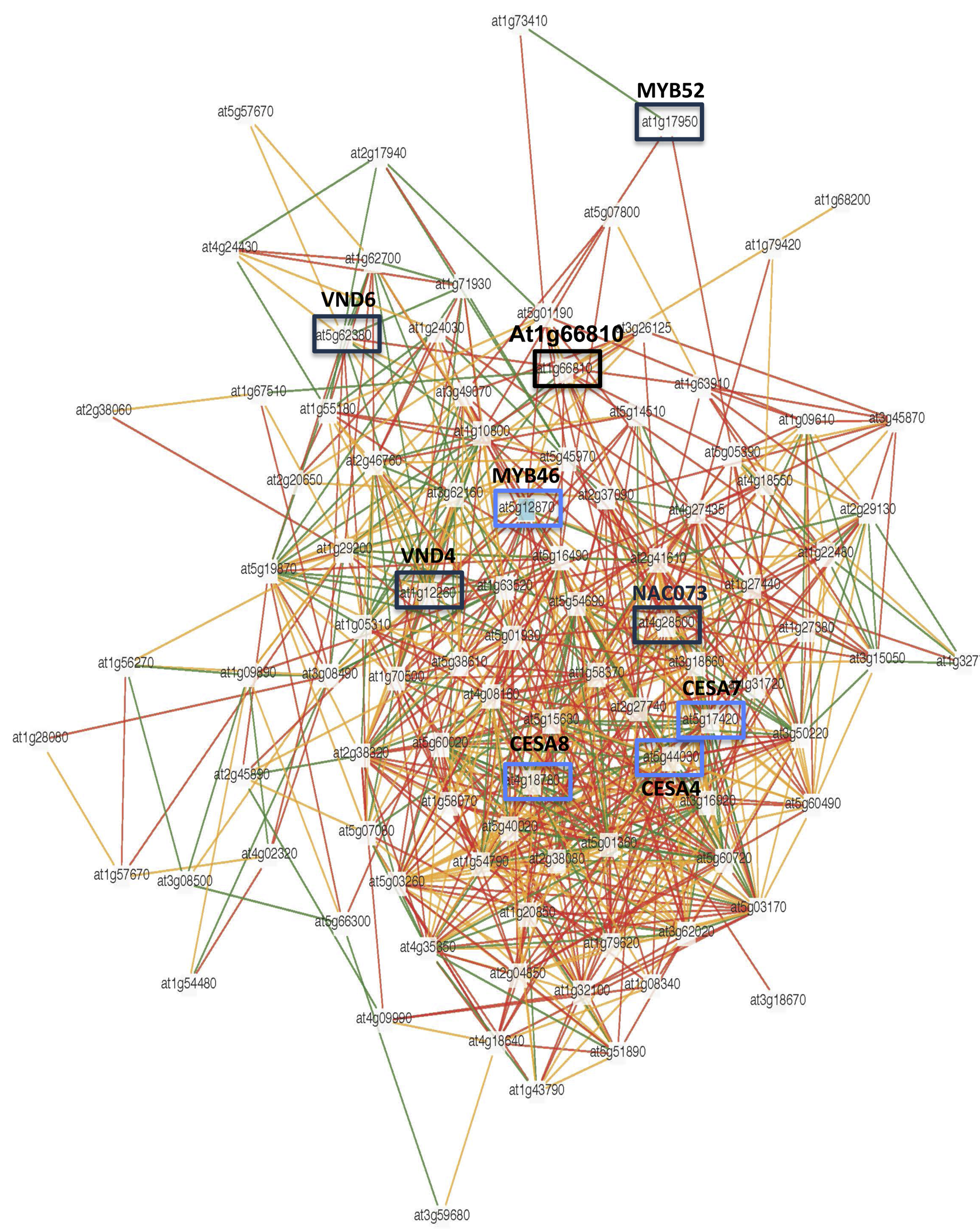

Supplement: S1 Fig — Nodes indicate individual genes, and edges indicate whether two genes are co-expressed above a certain mutual rank. The color edges indicate strength of the co-expression based on mutual rank relationships between the individual gene pairs. Green, orange, and red edges indicate a mutual rank relationship ≤10 (green), between 11 and 20 (orange) and 21 and 30 (red), respectively, for each connected gene. MYB46 and CESA4, CESA 7 and CESA8 encoding genes are highlighted and boxed in blue. The identified and co-regulated MYB46, VND6, At1g66810, VND4, and NAC073 transcription factor encoding genes are boxed in black. The network was generated, and modified from AraGenNet (http://aranet.mpimp-golm.mpg.de/aranet; Mutwil et al., 2010). (TIF) [file ppat.1004800.s001.tif]

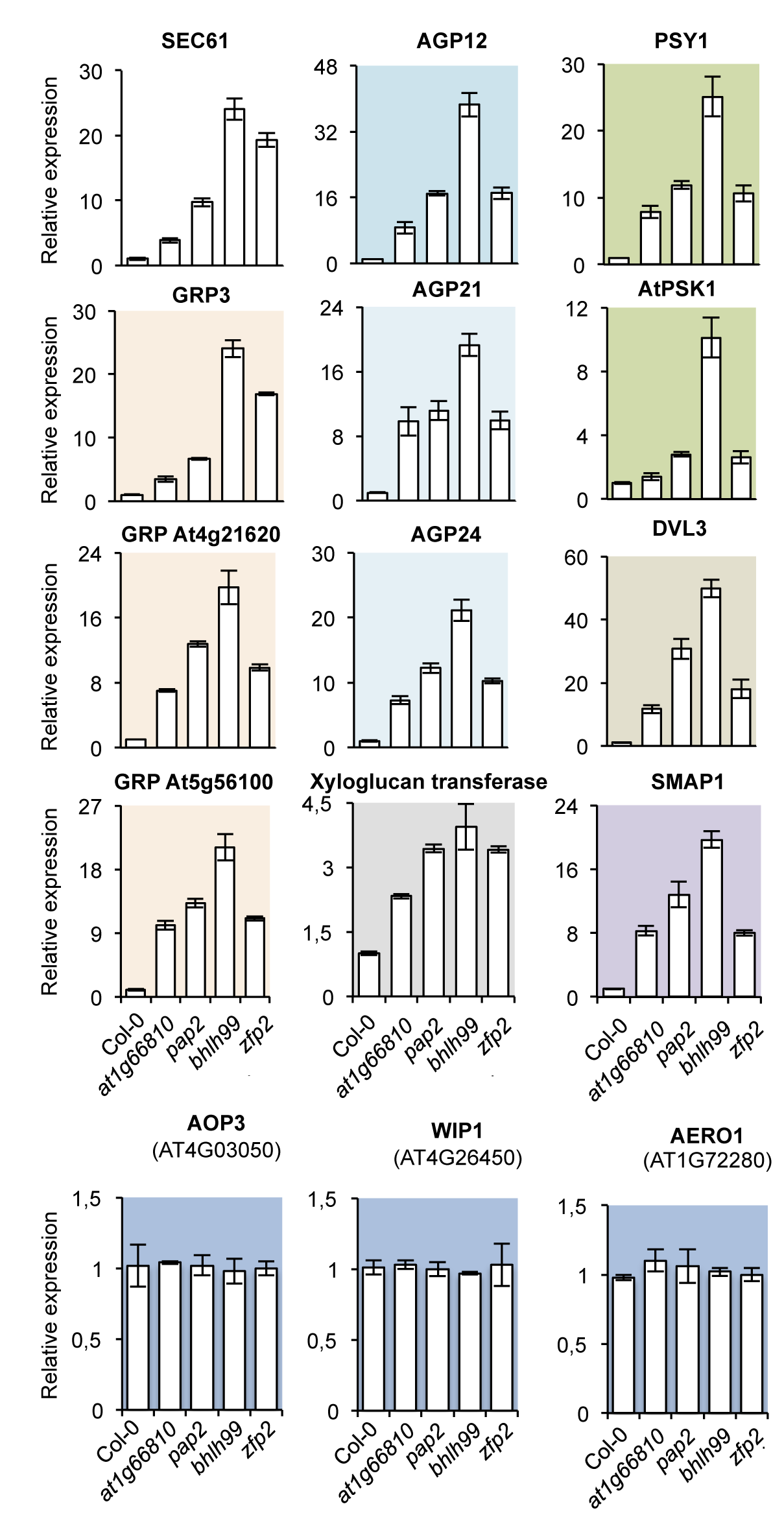

Supplement: S2 Fig — Expression levels of selected genes in Col-0 plants in comparison to at1g66810, pap2, bhlh99 and zfp2 mutants. Relative expression was assayed by quantitative RT-PCR on total mRNA isolated from leaves. Data represent mean ± SD (n = 3 biological replicates). Expression was normalized to the constitutive ACT2 gene. (TIF) [file ppat.1004800.s002.tif]

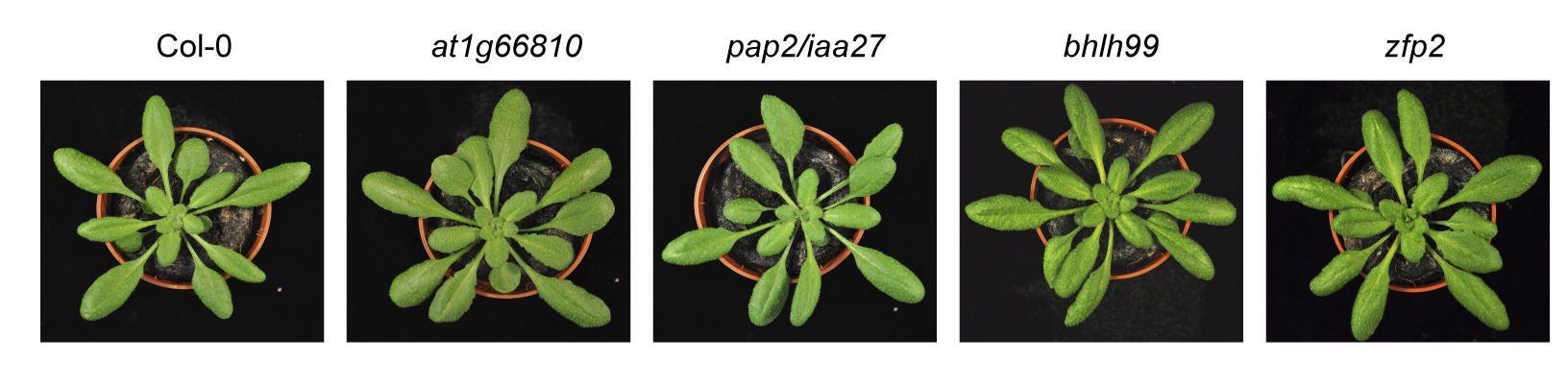

Supplement: S3 Fig — Plants were grown as indicated in Materials and Methods and pictures were taken at the vegetative stage of growth at 25 days after sowing. (TIF) [file ppat.1004800.s003.tif]

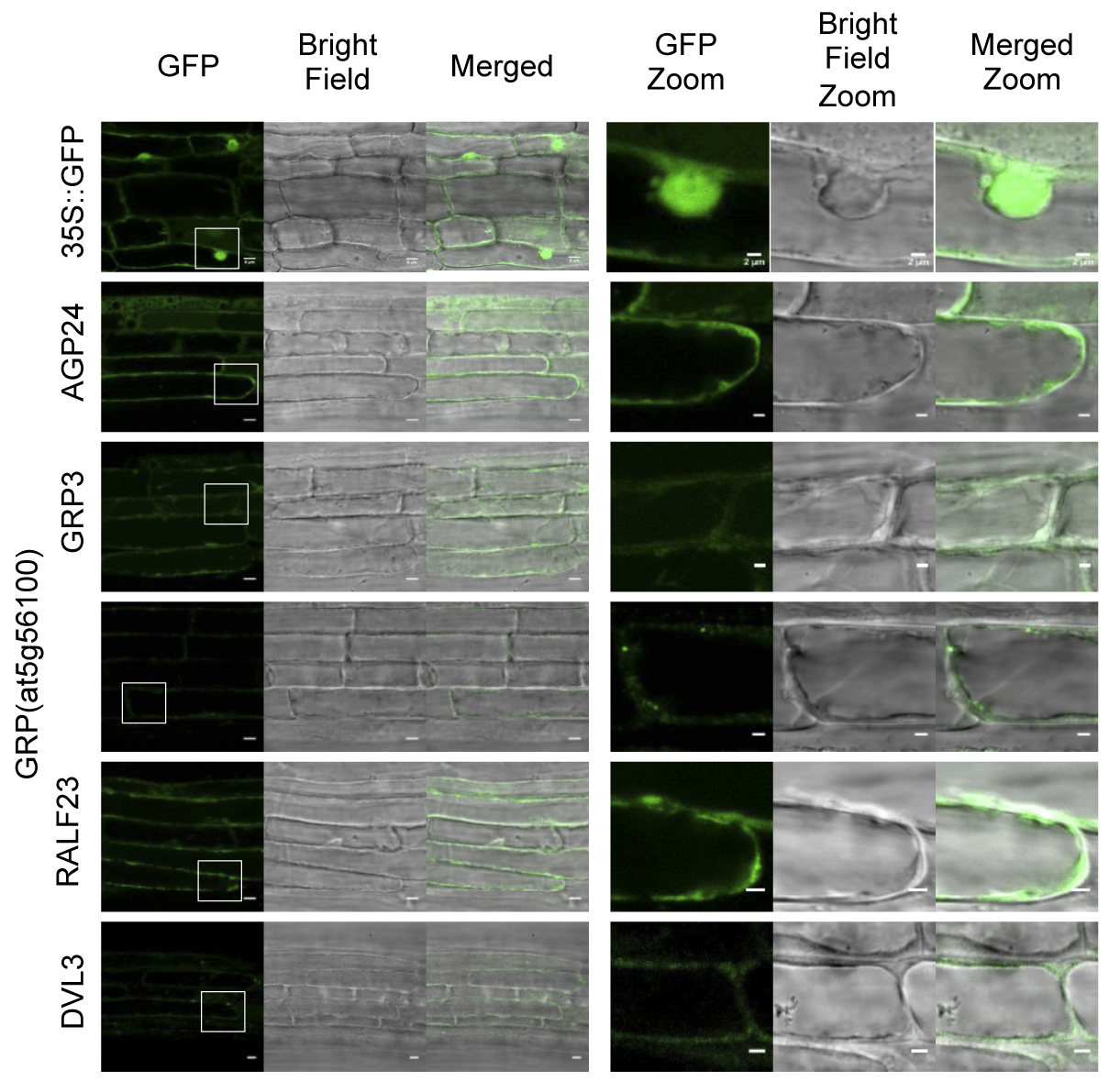

Supplement: S4 Fig — Left panel shows GFP localizations in epidermal cells of roots of transgenic plants expressing each fusion protein. Right panel shows a magnification of the tissue section shown in the left panel. Observe the intense nuclear localization of free GFP compare to the pericellular localization for each of the fusion proteins. (TIF) [file ppat.1004800.s004.tif]

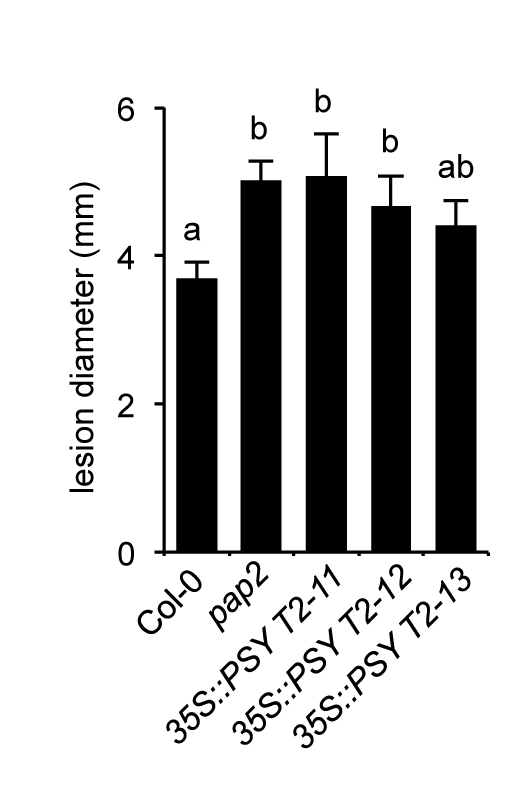

Supplement: S5 Fig — Lesion diameter of 20 plants per genotype and four leaves per plant were determined 11 d following inoculation with P. cucumerina. Values are means and ± SE (n = 80). ANOVA detected significant differences at the P < 0.05 level. (TIF) [file ppat.1004800.s005.tif]

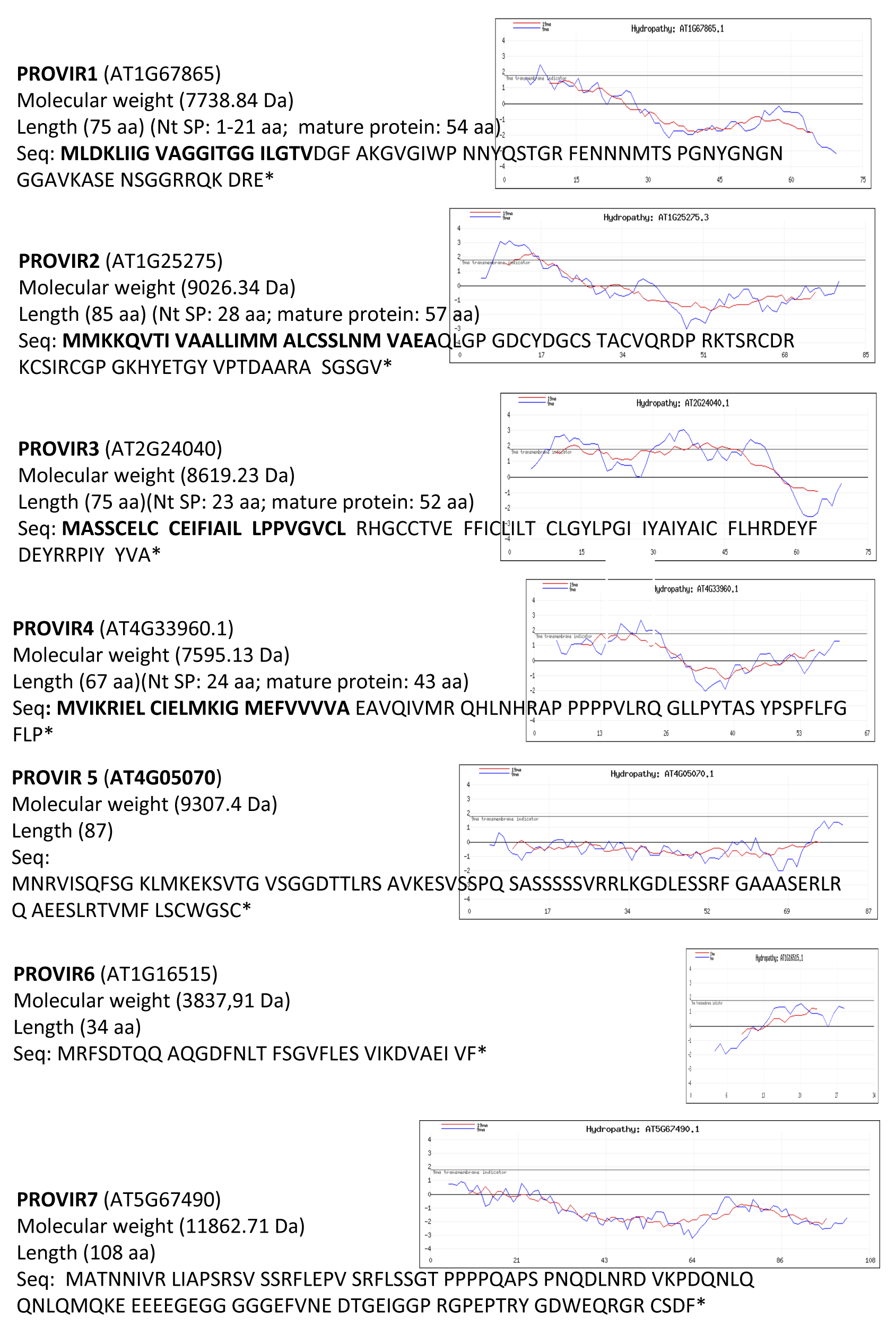

Supplement: S6 Fig — The theoretical molecular weight (Da) of the encoded protein, the length of the pre-protein, of its signal peptide (SP) and of the mature protein, in amino acids, is indicated in parenthesis. The amino acid sequence of the SP is indicated in bold. The hydropathy plot is indicated on the right of each amino acid sequence. (TIF) [file ppat.1004800.s006.tif]

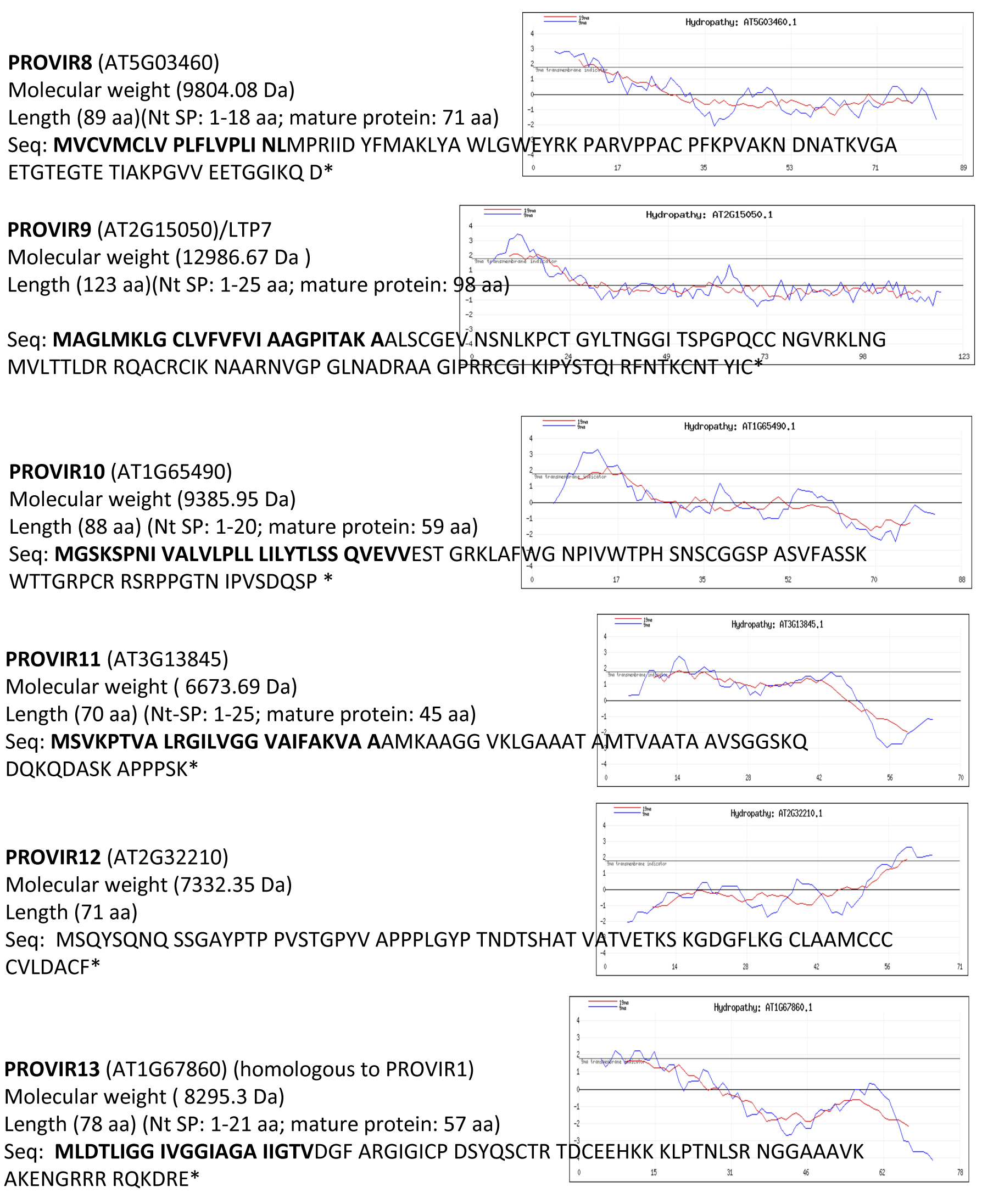

Supplement: S7 Fig — The theoretical molecular weight (Da) of the encoded protein, the length of the pre-protein, of its signal peptide (SP) and of the mature protein, in amino acids, is indicated in parenthesis. The amino acid sequence of the SP is indicated in bold. The hydropathy plot is indicated on the right of each amino acid sequence. (TIF) [file ppat.1004800.s007.tif]

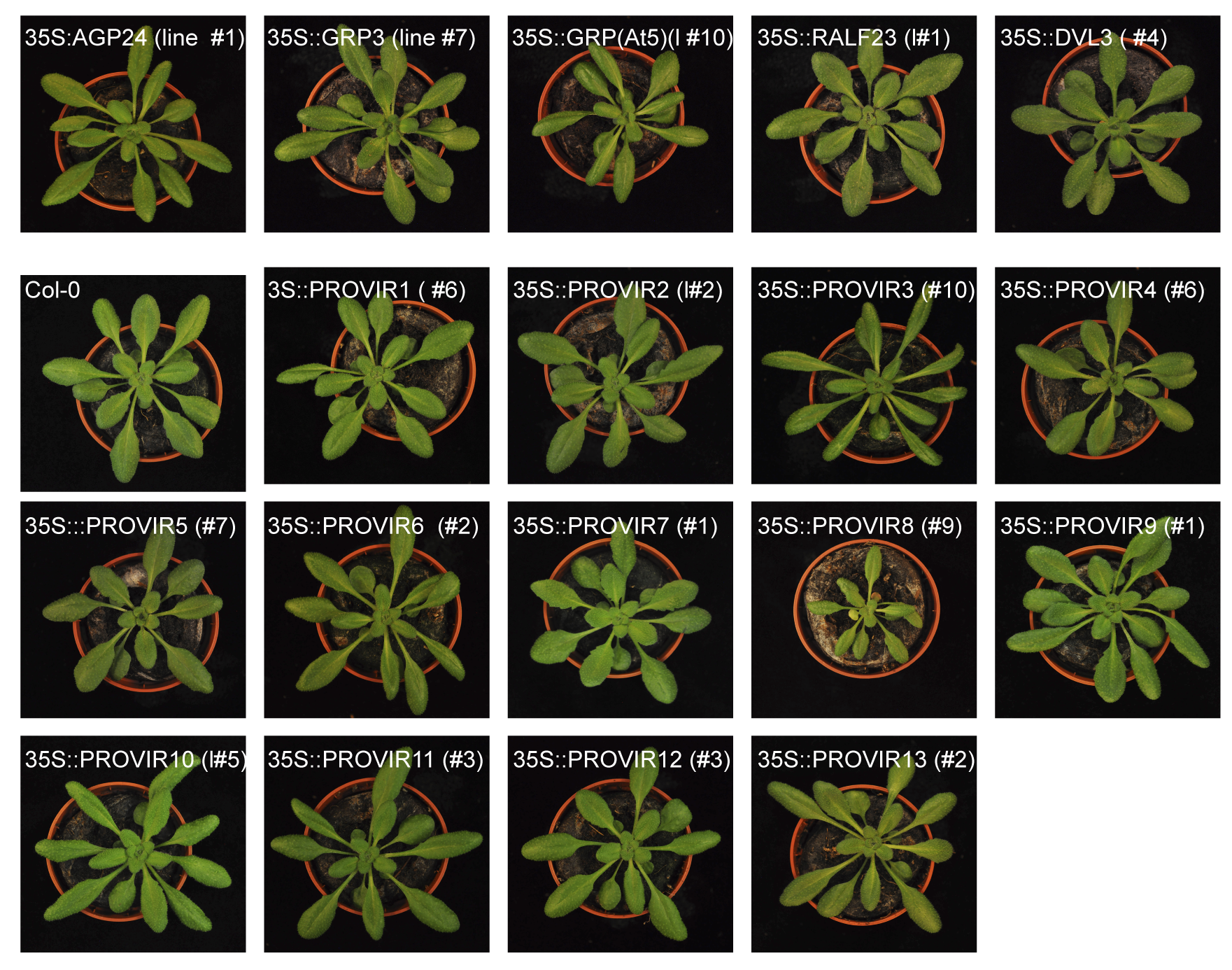

Supplement: S8 Fig — Plants were grown as indicated in Materials and Methods and pictures were taken at the vegetative stage of growth at 28 days after sowing. (TIF) [file ppat.1004800.s008.tif]

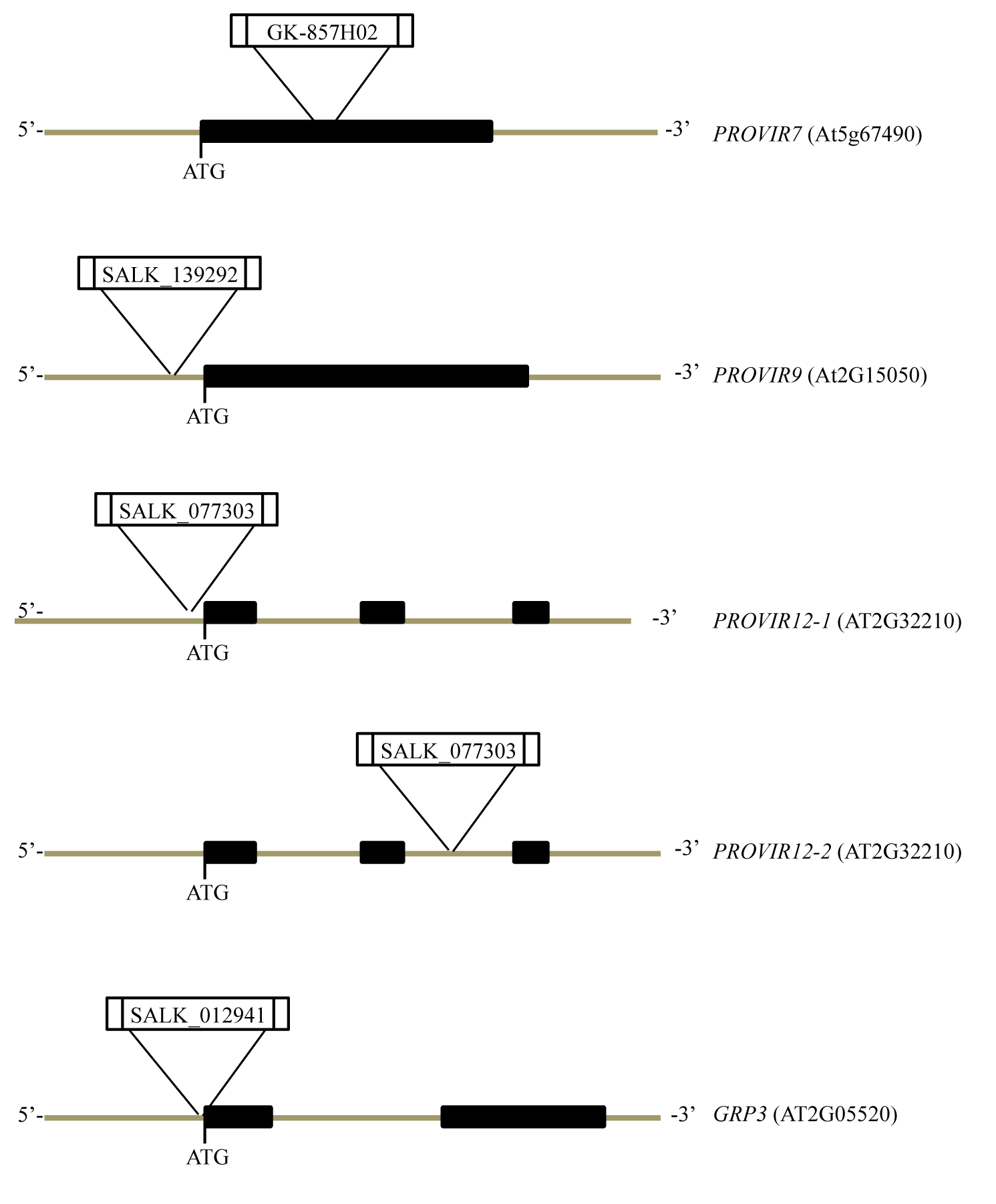

Supplement: S9 Fig — The provir7 mutant (strain GK-857H02) carries a T-DNA insertion internal to the unique exon and therefore disrupts the ORF. The provir9 mutant (strain SALK-139292) carries a T-DNA insertion upstream and proximal of the ATG initiation codon and therefore could affect expression of the gene. The provir12-1 mutant (strain SALK-077303) carries a T-DNA insertion upstream and proximal of the ATG initiation codon and therefore could affect expression of the gene. The provir12-2 mutant (strain SALK-077303) carries a T-DNA insertion internal to the second intron and therefore could alter mRNA stability and affect expression of the gene. The grp3 mutant (strain SALK-012941) carries a T-DNA insertion upstream and proximal of the ATG initiation codon and therefore could affect expression of the gene. Exons are indicated with solid rectangles. T-DNA insertions are indicated with white rectangles. (TIF) [file ppat.1004800.s009.tif]

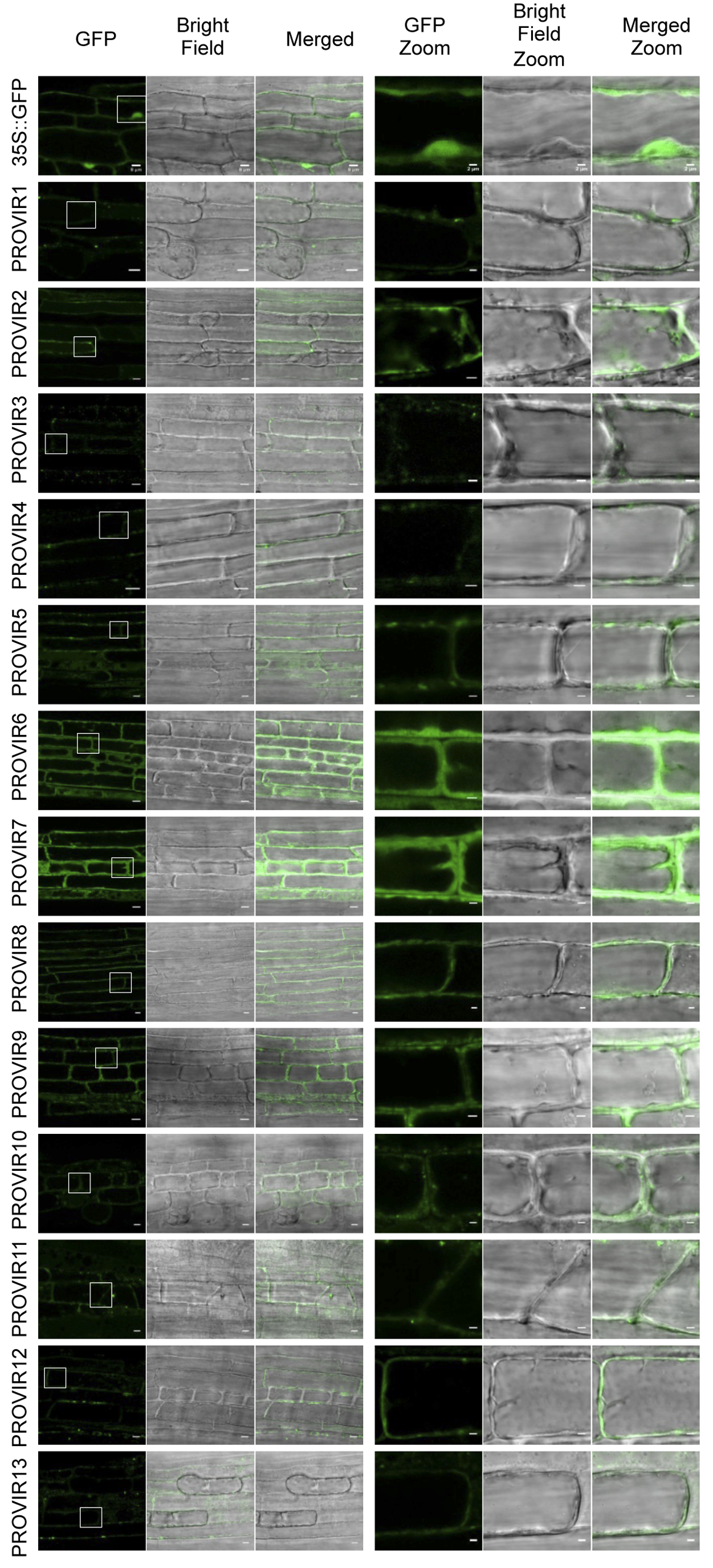

Supplement: S10 Fig — Left panel shows GFP localizations in epidermal cells of roots of transgenic plants expressing each fusion protein. Right panel shows a magnification of the tissue section shown in the left panel. Observe the intense nuclear localization of free GFP compare to the pericellular localization for each of the fusion proteins. (TIF) [file ppat.1004800.s010.tif]
