# Supplementary material for: Novel Disease Susceptibility Factors for Fungal Necrotrophic Pathogens in Arabidopsis
Source: PLoS Pathog. 2015 Apr 1;11(4):e1004800. doi: 10.1371/journal.ppat.1004800 (PMC4382300; doi:10.1371/journal.ppat.1004800)
Supplement: S1 Text — (DOCX) [file ppat.1004800.s015.docx]

*Relationship of primers used in this study.*

| Primer name | Nucleotide sequence (5` to 3`) | Use |
| --- | --- | --- |
| PROVIR1-BP-Fw | GGGGACAAGTTTGTACAAAAAAGCAGGCTATATGCTTGATAAGCTTATTATTGG | Cloning PROVIR1 in pDonr207 |
| PROVIR1-BP-Fw | GGGGACAAGTTTGTACAAAAAAGCAGGCTATATGCTTGATAAGCTTATTATTGG | Cloning PROVIR1 in pDonr207 |
| PROVIR2-BP-Rv-NO STOP | GGGGACAAGTTTGTACAAAAAAGCAGGCTATATGATGAAGAAGCAAGTGACG | Cloning PROVIR2 in pDonr207 |
| PROVIR2-BP-Fw | GGGGACCACTTTGTACAAGAAAGCTGGGTAAACACCACTCCCACTTGCC | Cloning PROVIR2 in pDonr207 |
| PROVIR3-BP-Fw | GGGGACAAGTTTGTACAAAAAAGCAGGCTATATGGCGAGTAGTTGCGAGC | Cloning PROVIR3 in pDonr207 |
| PROVIR3-BP-Rv-NO STOP | GGGGACCACTTTGTACAAGAAAGCTGGGTAAGCAACATAGTAGATTGG | Cloning PROVIR3 in pDonr207 |
| PROVIR4-BP-Rv-NO STOP | GGGGACAAGTTTGTACAAAAAAGCAGGCTATATGGTGATAAAAAGGATAG | Cloning PROVIR4 in pDonr207 |
| PROVIR4-BP-Fw | GGGGACCACTTTGTACAAGAAAGCTGGGTAAGGGAGAAATCCAAATAG | Cloning PROVIR4 in pDonr207 |
| PROVIR5-BP-Fw | GGGGACAAGTTTGTACAAAAAAGCAGGCTATATGAATAGAGTGATTAGTC | Cloning PROVIR5 in pDonr207 |
| PROVIR5-BP-Rv-NO STOP | GGGGACCACTTTGTACAAGAAAGCTGGGTAACAAGATCCCCAACAGCT | Cloning PROVIR5 in pDonr207 |
| PROVIR6-BP-Fw | GGGGACAAGTTTGTACAAAAAAGCAGGCTATATGAGGTTTAGTGATACAC | Cloning PROVIR6 in pDonr207 |
| PROVIR6-BP-Fw | GGGGACCACTTTGTACAAGAAAGCTGGGTAAAAAACAATTTCAGCGAC | Cloning PROVIR6 in pDonr207 |
| PROVIR7-BP-Rv-NO STOP | GGGGACAAGTTTGTACAAAAAAGCAGGCTATATGGCGACGAACAACATCG | Cloning PROVIR7 in pDonr207 |
| PROVIR7-BP-Rv-NO STOP | GGGGACCACTTTGTACAAGAAAGCTGGGTAGAAATCAGAGCATCGACC | Cloning PROVIR7 in pDonr207 |
| PROVIR8-BP-Rv-NO STOP | GGGGACAAGTTTGTACAAAAAAGCAGGCTATATGGTTTGCGTTATGTGTT | Cloning PROVIR8 in pDonr207 |
| PROVIR8-BP-Rv-NO STOP | GGGGACCACTTTGTACAAGAAAGCTGGGTAATCCTGCTTAATACCACC | Cloning PROVIR8 in pDonr207 |
| PROVIR9-BP-Rv-NO STOP | GGGGACAAGTTTGTACAAAAAAGCAGGCTATATGGCTGGTCTTATGAAGT | Cloning PROVIR9 in pDonr207 |
| PROVIR9-BP-Rv-NO STOP | GGGGACCACTTTGTACAAGAAAGCTGGGTAACATATGTACGTGTTGCA | Cloning PROVIR9 in pDonr207 |
| PROVIR10-BP-Rv-NO STOP | GGGGACAAGTTTGTACAAAAAAGCAGGCTATATGGGTTCAAAGTCACCAA | Cloning PROVIR10 in pDonr207 |
| PROVIR10-BP-Rv-NO STOP | GGGGACCACTTTGTACAAGAAAGCTGGGTATGGAGATTGATCAGAAAC | Cloning PROVIR10 in pDonr207 |
| PROVIR11-BP-Rv-NO STOP | GGGGACAAGTTTGTACAAAAAAGCAGGCTATATGTCGGTGAAACCCACAG | Cloning PROVIR11 in pDonr207 |
| PROVIR11-BP-Rv-NO STOP | GGGGACAAGTTTGTACAAAAAAGCAGGCTATATGTCGGTGAAACCCACAG | Cloning PROVIR11 in pDonr207 |
| PROVIR12-BP-Rv-NO STOP | GGGGACAAGTTTGTACAAAAAAGCAGGCTATATGAGTCAATACAGCCAAA | Cloning PROVIR12 in pDonr207 |
| PROVIR12-BP-Rv-NO STOP | GGGGACCACTTTGTACAAGAAAGCTGGGTAGAAGCATGCGTCGAGGAC | Cloning PROVIR12 in pDonr207 |
| PROVIR13-BP-Rv-NO STOP | GGGGACAAGTTTGTACAAAAAAGCAGGCTATATGCTTGATACGCTTATT | Cloning PROVIR13 in pDonr207 |
| PROVIR13-BP-Rv-NO STOP | GGGGACCACTTTGTACAAGAAAGCTGGGTACTCCCTGTCTTTCTGGCG | Cloning PROVIR13 in pDonr207 |
| AGP24-BP-Rv-NO STOP | GGGGACAAGTTTGTACAAAAAAGCAGGCTATATGATGATGATGACGAAGA | Cloning AGP24 in pDonr207 |
| AGP24-BP-Rv-NO STOP | GGGGACCACTTTGTACAAGAAAGCTGGGTAGTGGTTGGAACCAACGAC | Cloning AGP24 in pDonr207 |
| GRP3-BP-Rv-NO STOP | GGGGACAAGTTTGTACAAAAAAGCAGGCTATATGGCTTCCAAGGCTTTGG | Cloning GRP3 in pDonr207 |
| GRP3-BP-Rv-NO STOP | GGGGACCACTTTGTACAAGAAAGCTGGGTAGTGACCGGGCTGAGTCTG | Cloning GRP3 in pDonr207 |
| GRPX-BP-Rv-NO STOP | GGGGACAAGTTTGTACAAAAAAGCAGGCTATATGGAGAATCCTGGAGGAA | Cloning GRPX in pDonr207 |
| GRPX-BP-Rv-NO STOP | GGGGACCACTTTGTACAAGAAAGCTGGGTAAGAAGAAGAGTAATCCCG | Cloning GRPX in pDonr207 |
| RALF23-BP-Rv-NO STOP | GGGGACAAGTTTGTACAAAAAAGCAGGCTATATGAGAGGACTCTCCAGAA | Cloning RALF23 in pDonr207 |
| RALF23-BP-Rv-NO STOP | GGGGACCACTTTGTACAAGAAAGCTGGGTATGAGCGCCGGCAGCGAGT | Cloning RALF23 in pDonr207 |
| DVL3-BP-Rv-NO STOP | GGGGACAAGTTTGTACAAAAAAGCAGGCTATATGAAAGGTACCAAGAAGA | Cloning DVL3 in pDonr207 |
| DVL3-BP-Rv-NO STOP | GGGGACCACTTTGTACAAGAAAGCTGGGTAGTCATGCCAACAAATGAG | Cloning DVL3 in pDonr207 |

| at5g12870/MYB46 KO-FW | TGGCCTTCAATTCGACATTAG | *myb46* T-DNA mutant genotyping |
| --- | --- | --- |
| at5g12870/MYB46 KO-Rv | TGGTTTGCTTACAATTCTGGG | *myb46* T-DNA mutant genotyping |
| at4g28500/SND2/NAC73 KO-FW | ATAGGAGTGAACAAGGACGGG | *snd2/nac73* T-DNA mutant genotyping |
| at4g28500/SND2/NAC73 KO-Rv | AATAAACTTGTCGTTTGGGGG | *snd2/nac73* T-DNA mutant genotyping |
| at5g25830/GATA12 KO-FW | CATATTCAAGTTCGAGTGTCGC | *gata12* T-DNA mutant genotyping |
| at5g25830/GATA12 KO-Rv | CACTTTTATTGCGCTTGAAGG | *gata12* T-DNA mutant genotyping |
| at1g66810/AtC3H14 KO-FW | AGCTTTGCCTTAACCGTCTT | *at1g66810* T-DNA mutant genotyping |
| at1g66810/AtC3H14 KO-Rv | CCAAATCAATCTTTTCCCATG | *at1g66810* T-DNA mutant genotyping |
| at3g61910/NST2/ANAC066 KO-FW | TAAATAGGCCCCCACAAAATC | *nst2/anac066* T-DNA mutant genotyping |
| at3g61910/NST2/ANAC066 KO-Rv | CTTTGGTTTTGCCGACTTTTC | *nst2/anac066* T-DNA mutant genotyping |
| at4g29080/PAP2/IAA27 KO-FW | CTCTTTTCATGCTTCTGGTGG | *pap2/iaa27* T-DNA mutant genotyping |
| at4g29080/PAP2/IAA27 KO-Rv | TCCATGGTTGGAAACTCTGAC | *pap2/iaa27* T-DNA mutant genotyping |
| at5g65320/BHLH99 KO-FW | TTTTCACTCGTGGAAACATCC | *bhlh99* T-DNA mutant genotyping |
| at5g65320/BHLH99 KO-Rv | AAGGCGTCAATTGACAATCTG | *bhlh99* T-DNA mutant genotyping |
| at5g57520/ZFP2 KO-FW | AAGTGATGGTTTCAGCATGAAG | *zfp2* T-DNA mutant genotyping |
| at5g57520/ZFP2 KO-Rv | TCATCATCATGTTTGGAGCAG | *zfp2* T-DNA mutant genotyping |
| at3g49930 KO-FW | AAGTGTGAGTGCCAACCAATC | *at3g49930* T-DNA mutant genotyping |
| at3g49930 KO-Rv | AAAACGTACAAAACGTCACCG | *at3g49930* T-DNA mutant genotyping |
| at1g62360/STM KO-FW | CCTATTGACAAAAGCGAATCC | *stm* T-DNA mutant genotyping |
| at1g62360/STM KO-Rv | TTGATGTGATGTCATGATGGG | *stm* T-DNA mutant genotyping |
| at4g01680/MYB55 KO-FW | ACAAAAGGGTTTTTGTTTCGG | *myb55* T-DNA mutant genotyping |
| at4g01680/MYB55/BW52 KO-Rv | ATTGTGTGTTTTATGCCTGGC | *myb55* T-DNA mutant genotyping |
| at1g17950/MYB52/BW52 KO-FW | AAAAGGCTCTCATAAATAAAACCAC | *myb52/bw52* T-DNA mutant genotyping |
| at1g17950/MYB52 KO-Rv | TGGATGTACACTTGCATGTGG | *myb52/bw52* T-DNA mutant genotyping |
| at2g38090 KO-FW | TCTGAACATGTGTGACTTGCC | *at2g38090* T-DNA mutant genotyping |
| at2g38090 KO-Rv | CGATAATGGTGGAGCTCAAAC | *at2g38090* T-DNA mutant genotyping |
| at5g16600/MYB43 KO-FW | TTTGATTTTCGTGGTCCTCAC | *myb43* T-DNA mutant genotyping |
| at5g16600/MYB43 KO-Rv | ACTGTTGTTGAACTTGCCGTC | *myb43* T-DNA mutant genotyping |
| at5g62380/VND6/ANAC101 KO-FW | GTGGAAGAGGGACAGGAGAAG | *vnd6/anac101* T-DNA mutant genotyping |
| at5g62380/VND6/ANAC101 KO-Rv | TTGCTTATGTTTTTGGTTTTGC | *vnd6/anac101* T-DNA mutant genotyping |
| at1g12260/VND4/NAC007 KO-FW | AAAGGTCTCATCTGGATTATCCTG | *vnd4/nac007* T-DNA mutant genotyping |
| at1g12260/VND4/NAC007 KO-Rv | GGTGAAATCTAAAACCCGGAG | *vnd4/nac007* T-DNA mutant genotyping |
| at2g44745/WRKY12 KO-FW | TTGAACATCGAACTCATTTGTTG | *wrky12* T-DNA mutant genotyping |
| at2g44745/WRKY12 KO-Rv | GTCGTCAAGAACAGCCTTCAC | *wrky12* T-DNA mutant genotyping |
| at4g39410/WRKY13 KO-FW | TTAAAGCAAAACCGAGGTTCC | *wrky13* T-DNA mutant genotyping |
| at4g39410/WRKY13 KO-Rv | CGAACAAAACCAATCCAATTG | *wrky13* T-DNA mutant genotyping |
| at1g74660/MZF1 KO-FW | GGCGACGTACTATCAACATGC | *mzf1* T-DNA mutant genotyping |
| at1g74660/MZF1 KO-Rv | TTATAAAAGGGGCACAAAGGG | *mzf1* T-DNA mutant genotyping |
| at5g18090/MRG7.5 KO-FW | AACATCTCCGATCTCCAAACC | *mrg7.5* T-DNA mutant genotyping |
| at5g18090/MRG7.5 KO-Rv | CTTGAAACTTTGGCCTCAATG | *mrg7.5* T-DNA mutant genotyping |
| PROVIR7 KO-FW | CAGAACTCATGTAACCACCGG | *provir7* T-DNA mutant genotyping |
| PROVIR7 KO-Rv | TGGTTGATCGGAGATTGAGAG | *provir7* T-DNA mutant genotyping |
| PROVIR9 KO-FW | TTGTAGAGATCATGTGGATGTGG | *provir9* T-DNA mutant genotyping |
| PROVIR9 KO-Rv | ATGCATGACCATTGTCTGTTG | *provir9* T-DNA mutant genotyping |
| PROVIR12-1 KO-FW | CACTTTGGACGAGATAAAGCG | *provir12-1* T-DNA mutant genotyping |
| PROVIR12-1 KO-Rv | GACACAACAACAACACATGGC | *provir12-1* T-DNA mutant genotyping |
| PROVIR12-2 KO-FW | CAACGAATTAAAAGACCAAATCG | *provir12-2* T-DNA mutant genotyping |
| PROVIR12-2 KO-Rv | TGGATTCTAGGGCTTTATCGG | *provir12-2* T-DNA mutant genotyping |
| GRP3 KO-FW | GGTCCTGTAAATTTGCACCAC | *grp3* T-DNA mutant genotyping |
| GRP3 KO-Rv | GTATCGTCCTCCTCCTCCTTG | *grp3* T-DNA mutant genotyping |

| qRT-GRPAt1-Fw | CAAGCGGCAGCATGAATCTTCG | RT-qPCR |
| --- | --- | --- |
| qRT-GRPAt1-Rv | ACCGATAAAGCCAACCAACAGACC | RT-qPCR |
| qRT-GRPAt3-FW | GCCGCTTGAATGATGACCACTG | RT-qPCR |
| qRT-GRPAt3-Rv | AGCCACTTCCCATACCAAACGC | RT-qPCR |
| qRT-GRPAt4-Fw | TGGTGAGACCAACGGTTACTTGC | RT-qPCR |
| qRT-GRPAt4-Rv | CAGGACACCTAAGCTTCTTGCC | RT-qPCR |
| qRT-GRPAt5-Fw | AACGACCGGTTCTTCGGTTGTG | RT-qPCR |
| qRT-GRPAt5-Rv | AAAGCGGTCCTACCACTGCAAC | RT-qPCR |
| qRT-GRP3-Fw | TGGTTCTGTTGGGTCTCTTTGCTG | RT-qPCR |
| qRT-GRP3-Rv | TGAATTCACTGTGGCAGAAGATGC | RT-qPCR |
| qRT-GRPX-Fw | AACGACCGGTTCTTCGGTTGTG | RT-qPCR |
| qRT-GRPX-Rv | AAAGCGGTCCTACCACTGCAAC | RT-qPCR |
| qRT-XYLO-Fw | CTGGCTTGATAGAACCTCAGGAAG | RT-qPCR |
| qRT-XYLO-Rv | TGATGACTCCAGCAGTGTAGCC | RT-qPCR |
| qRT-AOP3-Fw | TTGCGGGAGATGCTCTATGTGC | RT-qPCR |
| qRT-AOP3-Fw | TACTCGGTGATACGGTGAAGGG | RT-qPCR |
| qRT-WIP1-Fw | GGGACCATCAAGAAGAGAGCATTC | RT-qPCR |
| qRT-WIP1-Rv | TGTTTCTAATGGGTCGTCGCTTTC | RT-qPCR |
| qRT-AERO1-Fw | GACGATGACACAGATAGTGGTGAG | RT-qPCR |
| qRT-AERO1-Rv | TGGAGTCCCAAATCCTTCTAGCTG | RT-qPCR |
| qRT-AGP12-Fw | TTTCTCCGCCGTAGGAAACGTG | RT-qPCR |
| qRT-AGP12-Rv | AGCAGCATCGGAAGTAGGACTTG | RT-qPCR |
| qRT-AGP21-Fw | TGGTTGTTGCGGTGGCTTTCTC | RT-qPCR |
| qRT-AGP21-Rv | GCAGCATCAGAAGTTGGGCTTG | RT-qPCR |
| qRT-AGP24-Fw | CCGTTGTTTCGGCCACCAATATG | RT-qPCR |
| qRT-AGP24-Rv | TGGTTGGAACCAACGACGAGAG | RT-qPCR |
| qRT-PSK1-Fw | AAGCTTGTGCCTGGCAGTTCTC | RT-qPCR |
| qRT-PSK1-Rv | AGTCCTCTTCCACTGATGTAGCTG | RT-qPCR |
| qRT-PSY1-Fw | CAACCCTGTTTCCGTTTCAGGTG | RT-qPCR |
| qRT-PSY1-Rv | CAACGTTCACCATCAACAAACTCC | RT-qPCR |
| qRT-PER42-FW | CGCTTTCTCTTGGCTCCGTAAC | RT-qPCR |
| qRT-PER42-Rv | AAAGCGACGCATCACATGACTC | RT-qPCR |
| qRT-RALF23-Fw | CGTCGCCGTATCTTCTCAATCCAC | RT-qPCR |
| qRT-RALF23-Rv | TTGTACCGCGGCACTCTGTTTC | RT-qPCR |
| qRT-RALF24-Fw | AAACGGCGAAATCGACGCAATG | RT-qPCR |
| qRT-RALF24-Rv | TCTCCGATGGCATCATCTCCTC | RT-qPCR |
| qRT-RALF32-Fw | AGGAATCAGCCTGCTTGCGATG | RT-qPCR |
| qRT-RALF32-Rv | AAGGGTTAGACGGTGGAGGAAGAC | RT-qPCR |
| qRT-DVL3-FW | AGGGATCGGATCTTCGTGGTTG | RT-qPCR |
| qRT-DVL3-Rv | CTCTATCTTGCGAACTCCGCTCAC | RT-qPCR |
| qRT-DVL6-FW | TGATGCTTAACCGGATCAAAGCG | RT-qPCR |
| qRT-DVL6-Rv | AGCCGGTTTCCTCCTTCGTATC | RT-qPCR |
| qRT-DVL10-Fw | GATGGCCAAACAGCAACGAACC | RT-qPCR |
| qRT-DVL10-Rv | AGCAGCATGGAGACACATCGTC | RT-qPCR |
| qRT-at1g66810-Fw | TCAACTCAGCTCTAACTCGGTGTC | RT-qPCR |
| qRT-at1g66810-Rv | CAACAAGCTCCAGTCTCTTGCC | RT-qPCR |
| qRT-PAP2/IAA27-Fw | TCTGAGGCTATCGGCTTAGCTC | RT-qPCR |
| qRT-PAP2/IAA27-Rv | TTTCCTAGTTCCTGCTTCTGCAC | RT-qPCR |
| qRT-BHLH99-Fw | AACGAAGACACAGCCAAGATTGC | RT-qPCR |
| qRT-BHLH99-Rv | TGACAATCTGCCTCTACCTTGACG | RT-qPCR |
| qRT-ZFP2-Fw | TCTCCGGTCGCTTTGAGCTTTAC | RT-qPCR |
| qRT-ZFP2-Rv | AGCCGCCACTTTCGAGAAATCC | RT-qPCR |
| qRT-PROVIR1-FW | ATGACGAGTCCGGGAAACTACG | RT-qPCR |
| qRT-PROVIR1-Rv | AGTTCTCGGATGCCTTCACTGC | RT-qPCR |
| qRT-PROVIR2-Fw | TCCAACGTGACCCGAGAAAGAC | RT-qPCR |
| qRT-PROVIR2-Rv | AGCATCTGGACCACAACGAATGG | RT-qPCR |
| qRT-PROVIR3-Fw | TCAGGCATGGCTGTTGCACTGTTG | RT-qPCR |
| qRT-PROVIR3-Rv | CGCGGTGCAAGAAACAAATTGCG | RT-qPCR |
| qRT-PROVIR4-Fw | TATGCGGCAACACCTCAACCAC | RT-qPCR |
| qRT-PROVIR4-Rv | AGGAATGGAGAAGGGTAAGAAGCG | RT-qPCR |
| qRT-PROVIR5-Fw | TCTGCCGTCAAAGAATCGGTCTC | RT-qPCR |
| qRT-PROVIR5-Rv | TTTAAGCCTCCTCACCGACGAC | RT-qPCR |
| qRT-PROVIR6-FW | ATGAGGTTTAGTGATACAC | RT-qPCR |
| qRT-PROVIR6-Rv | CAATTTCAGCGACATCTTTG | RT-qPCR |
| qRT-PROVIR7-Fw | ATGGCGACGAACAACATCG | RT-qPCR |
| qRT-PROVIR7-Rv | GTACCGGAGCTTAGAAACC | RT-qPCR |
| qRT-PROVIR8-Fw | AAACGACAACGCCACCAAAGTG | RT-qPCR |
| qRT-PROVIR8-Rv | CCTCTACAACGCCAGGTTTAGC | RT-qPCR |
| qRT-PROVIR9-Fw | AACCGTGCACTGGCTACTTGAC | RT-qPCR |
| qRT-PROVIR9-Rv | AGCTTTCTAACACCATTGCAGCAC | RT-qPCR |
| qRT-PROVIR10-Fw | AACGCAACGAAGAACGAAATGGG | RT-qPCR |
| qRT-PROVIR10-Rv | AACGCAAGTTTGCGCCCTGTAG | RT-qPCR |
| qRT-PROVIR11-FW | ACAAGCGCATTGACGGATTAGAC | RT-qPCR |
| qRT-PROVIR1-Rv | GCAGCTGCACCAAGCTTAACAC | RT-qPCR |
| qRT-PROVIR12-Fw | AGGTTACCCAACGAACGACACAAG | RT-qPCR |
| qRT-PROVIR12-Rv | ACAACACATGGCCGCAAGACAG | RT-qPCR |
| qRT-PROVIR13-Fw | ATTGGAACGGTGGATGGGTTCG | RT-qPCR |
| qRT-PROVIR13-Rv | AGTGCAGCTCTGGTAACTATCGG | RT-qPCR |
| qRT-PDF1.2a-Fw | CTTGTTCTCTTTGCTGCTTTC | RT-qPCR |
| qRT-PDF1.2a-Rv | CATGTTTGGCTCCTTCAAG | RT-qPCR |
| qRT-PR1-Fw | AAGGGTTCACAACCAGGCAC | RT-qPCR |
| qRT-PR1-Rv | CACTGCATGGGACCTACGC | RT-qPCR |
| qRT-ACT2-Fw | TCTTCCGCTCTTTCTTTCCAAGC | RT-qPCR |
| qRT-ACT2-Rv | ACCATTGTCACACACGATTGGTT | RT-qPCR |
